# A simple high-throughput method for automated detection of *Drosophila melanogaster* light-dependent behaviours

**DOI:** 10.1186/s12915-022-01476-z

**Published:** 2022-12-17

**Authors:** Thiago C. Moulin, Sovik Dey, Giovanna Dashi, Lei Li, Vaasudevan Sridhar, Tania Safa, Samuel Berkins, Michael J. Williams, Helgi B. Schiöth

**Affiliations:** 1 Department of Surgical Sciences, Functional Pharmacology and Neuroscience, Uppsala University, Uppsala, Sweden; 2 Department of Experimental Medical Science, Lund University, Lund, Sweden

**Keywords:** Light response, Jump response, Activity monitor, ON/OFF assay, Circadian rhythm, Ethanol intake, Spatial learning, Visual learning, Operant conditioning

## Abstract

**Background:**

Like most living organisms, the fruit fly *Drosophila melanogaster* exhibits strong and diverse behavioural reactions to light. *Drosophila* is a diurnal animal that displays both short- and long-term responses to light, important for, instance, in avoidance and light wavelength preference, regulation of eclosion, courtship, and activity, and provides an important model organism for understanding the regulation of circadian rhythms both at molecular and circuit levels. However, the assessment and comparison of light-based behaviours is still a challenge, mainly due to the lack of a standardised platform to measure behaviour and different protocols created across studies. Here, we describe the *Drosophila* Interactive System for Controlled Optical manipulations (DISCO), a low-cost, automated, high-throughput device that records the flies’ activity using infrared beams while performing LED light manipulations.

**Results:**

To demonstrate the effectiveness of this tool and validate its potential as a standard platform, we developed a number of distinct assays, including measuring the locomotor response of flies exposed to sudden darkness (lights-off) stimuli. Both white-eyed and red-eyed wild-type flies exhibit increased activity after the application of stimuli, while no changes can be observed in *Fmr1* null allele flies, a model of fragile X syndrome. Next, to demonstrate the use of DISCO in long-term protocols, we monitored the circadian rhythm of the flies for 48 h while performing an alcohol preference test. We show that increased alcohol consumption happens intermittently throughout the day, especially in the dark phases. Finally, we developed a feedback-loop algorithm to implement a place preference test based on the flies’ innate aversion to blue light and preference for green light. We show that both white-eyed and red-eyed wild-type flies were able to learn to avoid the blue-illuminated zones.

**Conclusions:**

Our results demonstrate the versatility of DISCO for a range of protocols, indicating that this platform can be used in a variety of ways to study light-dependent behaviours in flies.

**Supplementary Information:**

The online version contains supplementary material available at 10.1186/s12915-022-01476-z.

## Background

The *Drosophila melanogaster* model has been used for over a century to study a range of diverse biological processes and pathological conditions, as many of the major molecular mechanisms and signalling pathways that control physiological processes are similar to other organisms, including humans. Like many other insects, *Drosophila* shows strong behavioural responses to light. For instance, it exhibits phototaxis from its larval stage [1] to adult life [2]. Moreover, the fruit fly is a diurnal animal and has been a valuable model organism for understanding how circadian rhythms are regulated at the genetic, molecular, and neural circuit levels [3]. This response to light is important for the regulation of eclosion and courtship, determining the period of rest and activity, as well as modulating feeding times [4, 5]. The fly also shows rhythmic short-wavelength light avoidance, a behaviour considered crucial for avoiding damages caused by heat, low humidity, and ultraviolet radiation [6, 7]. Furthermore, they exhibit a complex pattern of light wavelength preference that changes according to the time of day [8].

Accordingly, many behavioural protocols are based on light stimuli [9, 10]. However, there is no standardised method or platform to systematically investigate the mechanisms underlying multiple light-dependent behaviours, which can lead to bias and inefficiency when using *Drosophila* as an experimental model. Therefore, in this study, we present a versatile high-throughput method for the assessment of behaviours based on light manipulations using a custom-made set-up we refer to as *Drosophila* Interactive System for Controlled Optical manipulations (DISCO). We describe how DISCO can be assembled using accessible components to measure a variety of behaviours. First, we assessed the flies’ visually evoked motor responses by presenting sudden darkness (lights-off) stimuli and assessing locomotion changes. We then investigate if this response can be inhibited by sequential stimuli presentation with short intervals. Next, to demonstrate the long-term use of DISCO, we analyse alcohol preference patterns over the course of 48 h. Finally, we implemented a feedback-loop algorithm to develop a place preference task based on the flies’ innate wavelength-specific light avoidance. We conclude that DISCO can be used for high-throughput assessment of various light-dependent behaviours, generating results that can contribute to many research fields beyond *Drosophila* neurophysiology, such as translational genomic and pharmacological screenings of behavioural outcomes.

## Results

### Light manipulation and locomotion detection by the DISCO apparatus

**Fig. 1:**
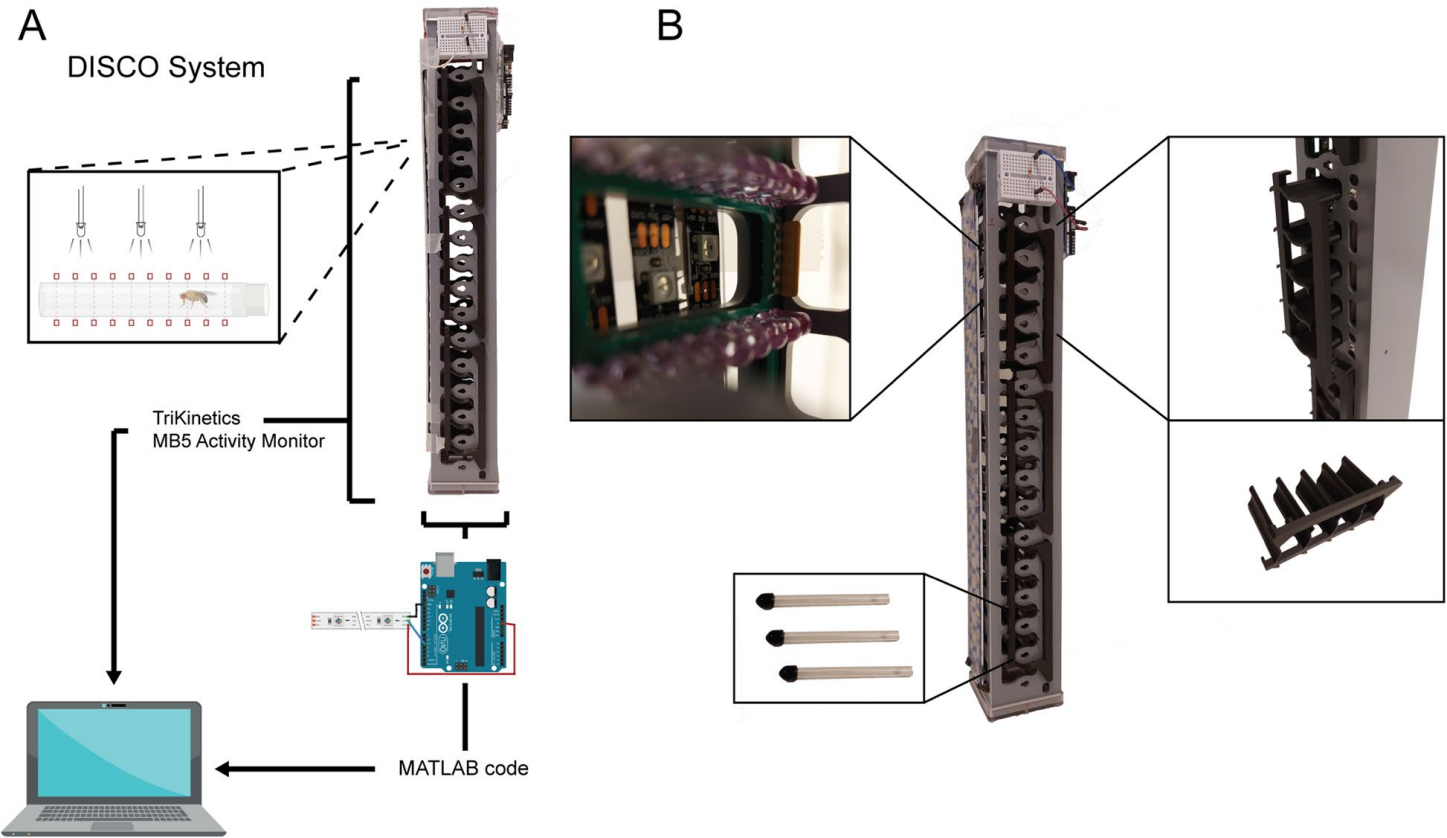
Schematic representation of the DISCO system. **A** Integration of the MB5 infrared-based device for activity assessment and RGB LED lights controlled by an Arduino/MATLAB interface. The system allows for millisecond-resolution control of LED lights, while recording locomotion and position for each second. The MATLAB interface can also use the activity reads as input, enabling feedback loop protocols. **B** Representation of the main components of DISCO: infrared beams, LED illumination, and tube slots barriers for individual light manipulation

To develop an automated method for light-based tasks that could reduce the variability caused by subjective assessments, we produced the DISCO system. We employed a commercially available activity monitor based on infrared detectors, which can detect the movements of flies sequestered in individual glass tubes (MB5 Multi-Beam Activity Monitor, Trikinetics). Importantly, the activity monitor chosen has 17 independent infrared beams, so the unit was able to record any movement at any location within the length of each tube. LED light stripes were coupled to the device, which was manipulated by a programmable Arduino microcontroller and custom-made MATLAB software (Fig. 1A). In the device, each tube was illuminated by three LED lights, and each tube slot was separated by custom-made 3D-printed barriers (Fig. 1B). The MATLAB-Arduino interface allowed for the designing of protocols tailored for each experiment with millisecond-precise light presentations, while keeping an accurate record of stimuli timing, necessary for behavioural analyses.

### Effects of transient sudden darkness stimuli on locomotion

A protocol was designed to assess motor responses after the presentation of visual stimuli, in this case, transitory darkness perceived by the fly as the shadow of a nearby predator. Current methods measure the flies’ escape reaction by the presentation of a transient shadow or looming stimuli, evoking a jump response, especially in white-eyed flies [11, 12]. However, we qualitatively verified that presenting sudden darkness (lights-off) for a second, while not able to induce a jump response in our sample, did induce an increase in locomotion, even in red-eyed wild-type flies (Additional file 5: Video S1).

**Fig. 2:**
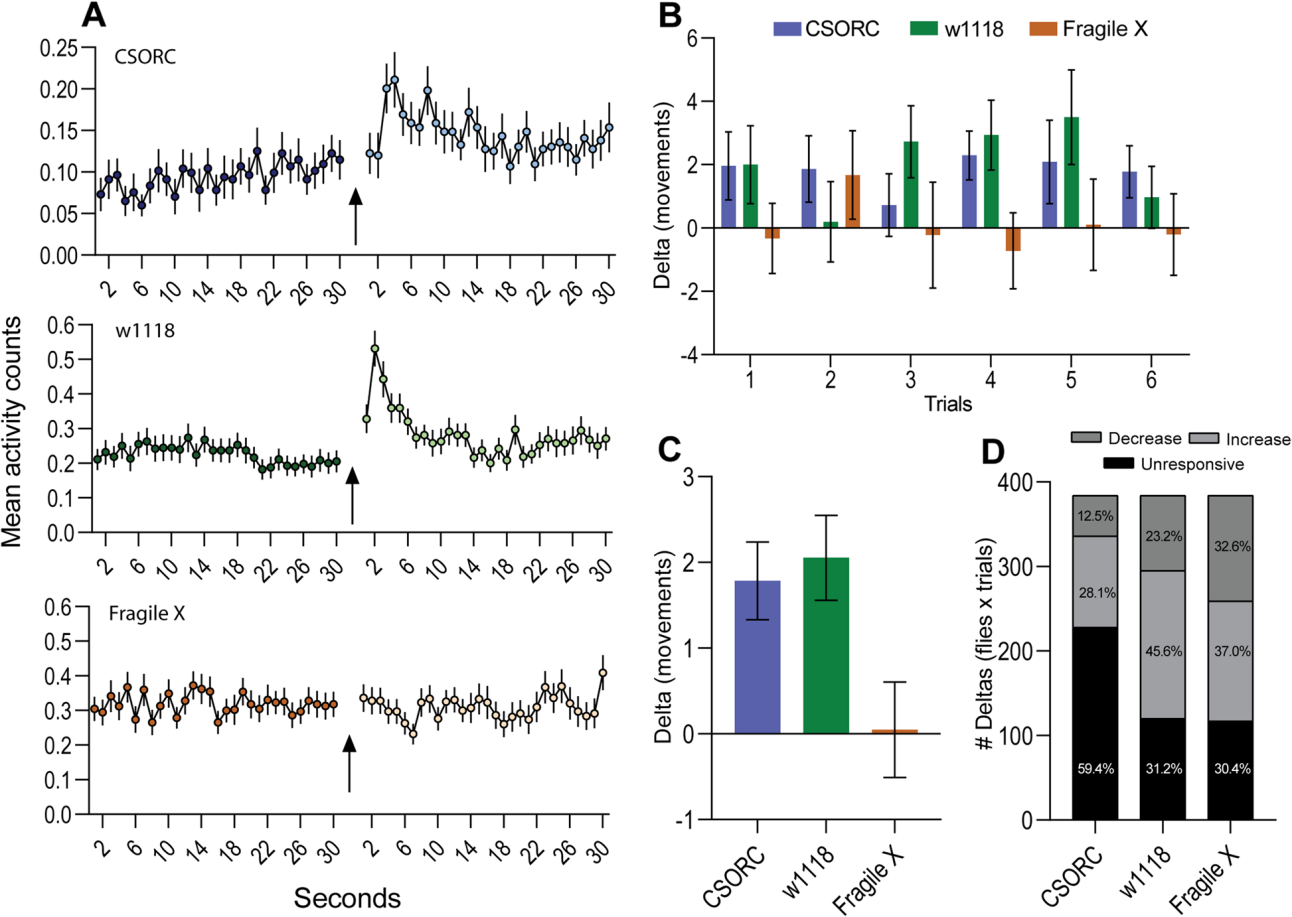
Effects of 1-s darkness stimuli on locomotion. **A** Second-by-second average activity of the flies before and after the sudden darkness stimuli for all trials. **B** Delta index was calculated for each stimuli presentation trial (*p* = 0.011 for lines’ differences; two-way ANOVA). **C** Average of all trials’ delta index (*CSORC* × Fragile X *p* = 0.033; *w*^*1118*^ × Fragile X *p* = 0.015; one-way ANOVA and Holm-Sidak post hoc test). **D** Percentages of trials in which flies responded with a decrease, increase, or no change in movements (*n*_CSORC_ = 58; *n*_w1118_ = 62; *n*_Fragile X_ = 58 from 4 independent experiments)

To systematically measure the flies’ change in movement, DISCO presented to the flies a constant white light illumination for 30 min, which was briefly turned off for 1 s, before restarting the stimulation loop. This procedure was repeated for 3 h (six trials). Three genetic fly lines were tested: *w*^*1118*^ (white-eyed), *CSORC* wild type (red-eyed), and fragile X syndrome fly models (*Fmr1* null allele, white-eyed). The *w*^*1118*^ and *CSORC*, but not the fragile X, flies presented a movement increase in response to the sudden darkness stimuli (Fig. 2A). For comparison between the groups, we calculated the delta index (average movement after stimuli presentation minus the average movement before stimuli presentation). Two-way ANOVA of the trials’ delta indexes (Fig. 2B) indicated a significant difference between lines (*p* = 0.011). Further comparison of the overall trials’ average delta indexes (Fig. 2C) showed a significant difference between fragile X flies vs *CSORC* (*p* = 0.033) and *w*^*1118*^ flies (*p* = 0.015). No differences in the locomotion response index were found between males and females (Additional file 1: Fig. S1).

Further investigation of the response from single flies shows that, despite a clear average group effect, individual darkness-induced locomotion was highly variable (Additional file 2: Fig. S2 A). After stimuli presentation, although mostly positive, movements were reduced for a noticeable number of flies, exhibiting a wide range of delta scores (min − 39, max 37 movements across six trials). Thus, we analysed the percentages of trials in which flies responded with a decrease, increase, or no change in movements (Fig. 2D). *CSORC* wild-type flies were mostly unresponsive to the stimuli; however, sudden darkness induced a movement increase in most cases when responsive. The same pattern was seen for *w*^*1118*^ flies, although this line was much more responsive to the stimuli. Fragile X flies were also highly responsive but exhibited similar levels of increased and decreased movements after stimuli.

Paired plots and two-way ANOVA analyses of movement counts before and after stimuli (Additional file 2: Fig. S2B-D) confirmed a significant increase in activity after transient darkness presentation for both CSORC and *w*^*1118*^ groups (*p*_CSORC_ = 0.0002 and *p*_w1118_ = 0.0001), but not for fragile X flies (*p* = 0.9303). We also observed a significant difference in the individual flies’ behaviour across trials for all lines (*p*_CSORC_ = 0.0419; *p*_w1118_ = 0.0429; *p* fragile X = 0.0489), indicating that the amplitude of one’s locomotor response is prone to trial-by-trial variations. Analyses of the correlation between locomotion values before and after stimuli for all trials show that flies with higher activity previous to transient darkness presentation also show increased activity after stimuli (Additional file 3: Fig. S3). This was true for CSORC (*r* = 0.501; *p* < 0.0001), *w*^*1118*^ (*r* = 0.559; *p* < 0.0001), and fragile X flies (*r* = 0.476; *p* < 0.0001), suggesting that, in general, higher values of locomotion after stimuli can be seen from already active flies, independent of an overall group increase or decrease in response.

Lastly, we verified if the observed stimulation effects in the locomotion response from fragile X flies to CSORC and *w*^*1118*^ controls could be attributed to different baseline activities by comparing the average activity counts and speed for the 20 min preceding the first-trial stimulus (Additional file 4: Fig. S4). One-way ANOVA showed that CSORC flies were significantly less active than *w*^*1118*^ (*p* = 0.0005) and fragile X flies (*p* = 0.001), while the *w*^*1118*^ and fragile X groups were comparable (*p* = 0.727). Additionally, the average baseline speed of fragile X flies was significantly bigger than CSORCs (*p* = 0.003) but smaller than *w*^*1118*^ controls (*p* = 0.011). Overall, our analysis indicates that the activity of the fragile X line was similar to the highly responsive *w*^*1118*^ flies, and their baseline speed lies between the range of the two control lines.

### Inhibition of sudden darkness-induced locomotion by repeated stimuli presentation

**Fig. 3:**
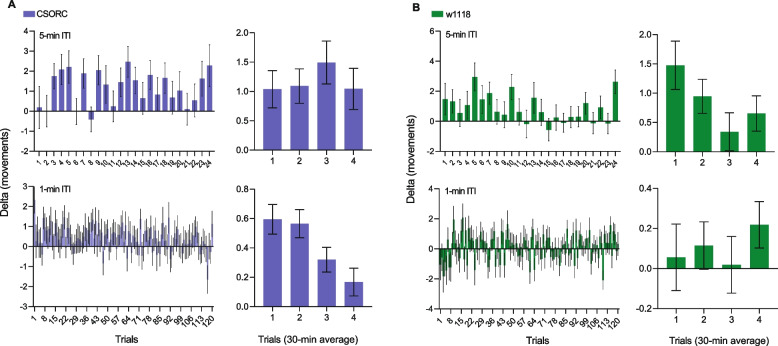
Effects of repeated sudden darkness presentation. For 2 h, flies underwent stimuli presentation with a 5-min (upper panels) or 1-min (bottom panels) intra-trial interval (ITI). For analysis, we grouped and averaged the trials’ delta outcomes in 30-min recording epochs, containing *n* = 6 and *n* = 30 delta values each for 5 and 1, respectively (right panels). **A** *CSORC* wild-type flies showed no reduction of movements with 5-min ITI (*p* = 0.779, slope = 0.042, *R*^2^ = 0.060) but exhibit a significant linear trend with the 1-min ITI protocol (*p* = 0.0003, slope = − 0.153, *R*^2^ = 0.932). **B** The *w*^*1118*^ line showed a significant reduction trend for 5-min ITI (*p* = 0.041, slope = − 0.307, *R*^2^ = 0.674), while no difference was found between trials for the 1-min ITI protocol (*p* = 0.522, slope = 0.039, *R*^2^ = 0.338). All analyses were performed on 30-min epoch average values using one-way ANOVA and post hoc test for linear trend (*n* = 96/group from 3 independent experiments)

Next, we altered the stimuli protocol to verify if the repeated presentation of 1-s sudden darkness would alter the locomotion response of the flies. We tested two distinct protocols, one where flies underwent sudden darkness stimuli presentation with a 5-min inter-trial interval (ITI) and another with 1-min ITI. We observed that *CSORC* wild-type flies did not change their locomotion pattern after stimuli presentation at 5-min ITI (*p* = 0.779) but significantly reduced their activity with time at 1-min ITI (*p* = 0.0003) (Fig. 3A). On the other hand, *w*^*1118*^ flies were more sensitive to repeated stimuli presentation, exhibiting a significant downward trend of activity during the 5-min ITI protocol (*p* = 0.041). Additionally, the *w*^*1118*^ line presented a reduced locomotion response from the first trial at the 1-min ITI, thus showing no significant temporal trend of activity reduction, likely due to a floor effect (*p* = 0.522) (Fig. 3B). These results indicate that sudden darkness-induced locomotion can be inhibited with multiple stimuli presentation, however, with different sensitivity between red-eyed and white-eyed flies.

### Combined analysis of alcohol preference and circadian rhythms

To demonstrate the long-term use of DISCO, we developed a protocol of food preference where each individual tube had two food options. On one end, low-sugar food (5% sucrose, 5% yeast, and 2% agarose) was placed, while at the other end, there was the same food with 15% ethanol added. Preference was measured in terms of the percentage of time spent at the end of the tubes, measured by each side’s last two infrared beams. DISCO was programmed to a 12:12 light-dark cycle, and the overall activity of the flies was also recorded for 48 h. Male and female *CSORC* flies were used and analysed separately.

**Fig. 4:**
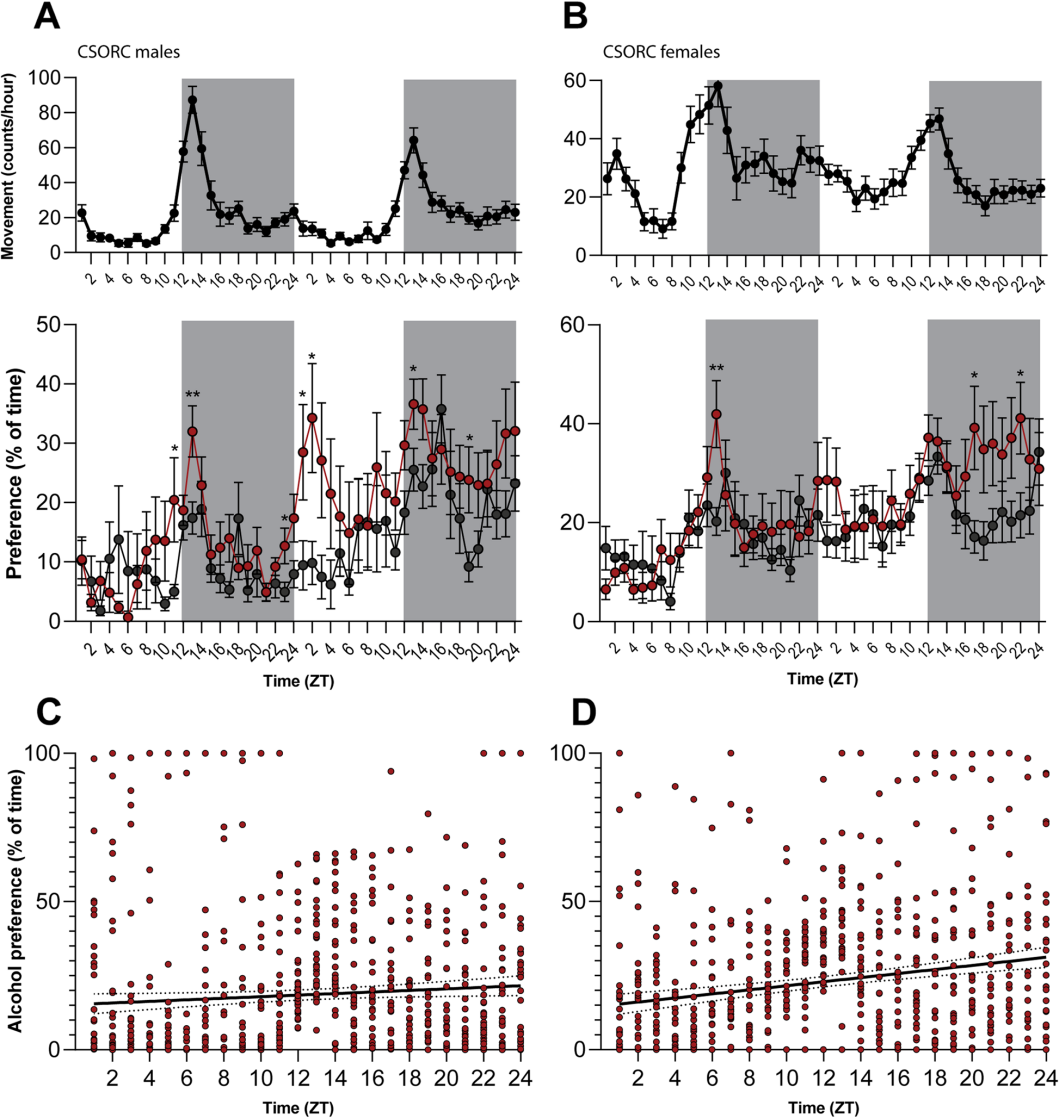
Alcohol preference through time. **A** *CSORC* male and **B** female flies were monitored for 48 h in a 12:12 dark-light cycle, and the time spent at the tube ends was recorded. Differences between normal food (grey lines) and alcoholic food consumption (red lines) were analysed by a two-way ANOVA followed by an LSD post hoc test. *p < 0.05 and **p < 0.01 **C**, **D** Correlation between alcoholic food preference values and ZT times for males (*r* = 0.078, *p* = 0.038) and females (*r* = 0.201, *p* < 0.0001) (*n*_males_ = 15; *n*_females_ = 14 from 2 independent repeats each)

We observed that the preference for alcoholic food was not constant throughout the days. Rather, the choice for ethanol happened intermittently for both male and female flies, and not always following peaks of motor activity (Fig. 4A, B). Then, we tested if there were any trends in ethanol consumption depending on the time of day by correlating the alcoholic food preference values with the ZT times. We found a positive correlation between those variables for male (*r* = 0.078, *p* = 0.038) (Fig. 4C) and female (*r* = 0.201, *p* < 0.0001) flies (Fig. 4D), indicating a small but significant trend for alcohol consumption in dark phases of the day.

### Place preference induced by different light frequencies through feedback-loop manipulation

Lastly, we sought to implement a feedback loop system that presented different stimuli depending on the flies’ position. We developed a place-preference test exploring *Drosophila*’s aversion to blue light and preference for green light [8]. The recordings from the MB5 equipment were read live by a MATLAB routine which also controlled the light stimuli. The tubes were divided into two zones, and when the flies crossed to one of those specified positions of the tube (e.g. the left zone), either blue or green light stimuli were presented. The preference was then measured by the time spent under the blue- or green-illuminated zones.

**Fig. 5:**
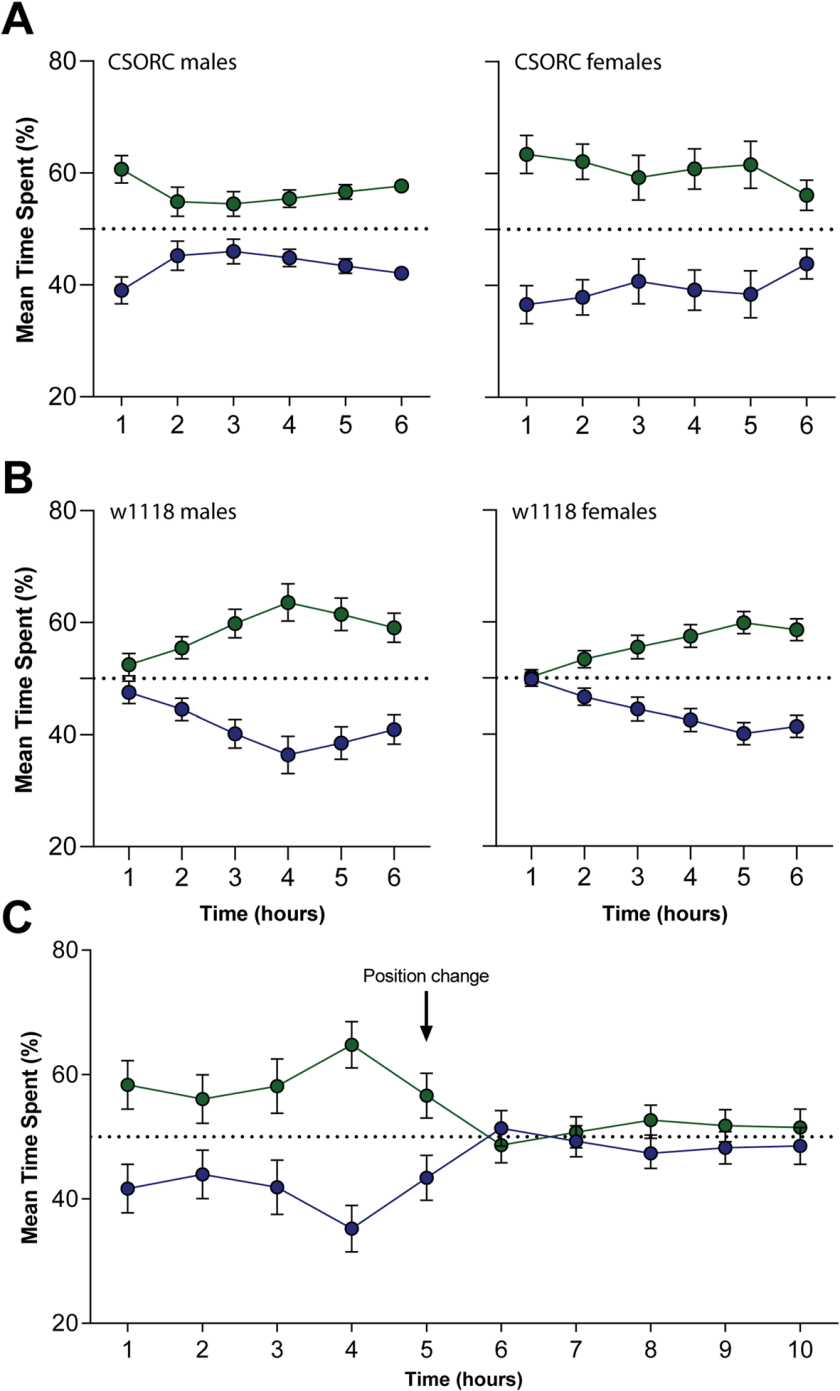
Light-dependent place preference. **A** Male and female CSORC flies show blue light avoidance behaviour from the first hour of the trial (*p*_males_ < 0.0001, *p*_females_ = 0.0005; two-way ANOVA; *n*_males_ = 80, *n*_females_ = 31 from 5 and 2 independent repeats, respectively). **B** The w1118 group also avoid blue light exposure (*p*_males_ < 0.0001, *p*_females_ < 0.0001; two-way ANOVA; *n*_males_ = 48, *n*_females_ = 64 from 3 and 4 independent repeats, respectively); however, they exhibit a gradual learning curve. **C** Male CSORC flies were tested for 10 h, where at hour 5, the blue- and green-illuminated zones were interchanged, inducing the loss of place preference behaviour (*n* = 96 from 3 independent repeats)

We observed that both *CSORC* male and female flies successfully learned to avoid the blue-illuminated zones (*p*_males_ < 0.0001, *p*_females_ = 0.0005). The blue light avoidance could be seen already from the first hour and was maintained through the conditioning session (Fig. 5A). The *w*^*1118*^ flies were also able to learn the task (*p*_males_ < 0.0001, *p*_females_ < 0.0001); however, their learning curve was gradual along the course of the session (Fig. 5B). Finally, to verify if the task involved place-preference learning and not just blue light avoidance responses, the test was repeated with CSORC males for 10 h, where at the 5th hour, the illumination zones were interchanged (e.g. the left zone that showed blue light stimuli starts to present green light stimuli). We observed that, as before, the CSORC flies learned to avoid blue light zones from the first hour of the trial, but after the stimuli zones were changed, the zone preference times stayed at the chance level (Fig. 5C).

## Discussion

### The DISCO device

In this study, we demonstrate a new method for investigating light-based behaviours in *Drosophila* by merging widely used equipment for locomotion monitoring and a computationally controlled light system into an apparatus named the *Drosophila* Interactive System for Controlled Optical manipulations (DISCO). We based our device on the commercially available MB5 activity monitor; however, DISCO modifies the existing infrared-based detection of locomotion by adding a robust, yet straightforward, custom set-up for light-controlled interventions. In fact, our objective was to create simple modifications that can be easily adapted to different activity monitors according to the experimental need, while providing a flexible platform for a range of protocols.

We achieved this goal by integrating RBG-LED lights into the motion detector device, while managing the lights’ function by implementing a MATLAB-Arduino system. These steps, which are easily reproducible, allow us to (i) create complex lights on/off protocols with millisecond precision, (ii) investigate light-based behaviours during short- and long-time monitoring, (iii) study the effects of different light frequencies on *Drosophila* activity, and (iv) develop second-by-second feedback loop protocols where the motion or position of the flies can modulate the light intervention.

Due to such versatility, the DISCO platform can be used in a variety of ways to study light-dependent behaviours in *Drosophila*. For instance, in addition to the protocols presented here, it may be used to study light-based appetitive and aversive behaviours, as well as for optogenetic studies. Moreover, the use of the platform for long-term experiments allows for the analysis of circadian responses. Due to the easy device assembly and the possibility of testing multiple individual flies, DISCO can be easily employed for high-throughput experimental settings, which can significantly contribute to drug screenings or genomic studies.

### Sudden darkness-induced locomotion

The responses of *Drosophila* to light-dark stimuli are studied in a range of paradigms. In *Drosophila* larvae, protocols with intermittent light and dark pulses, also called ON/OFF assays, have been used to measure several locomotory parameters. Larvae show an increase in the distance travelled on the lights-off onset while displaying more head swinging and change of direction behaviours on lights-on onset [13, 14]. This behaviour is shown to be independent of the neuronal circuitry underlying circadian rhythmicity [15]. Notably, the response to light is abolished during the transition to mid-larval third instar, where there is a change from foraging to wandering period, as the fly searches for a site for metamorphosis [16].

In adult flies, the presentation of sudden darkness (lights-off) stimuli triggers a jump response, known to be mediated by the pair of giant descending neurons, also called giant fibres, which convey visual and mechanosensory information to the thoracic ganglia neurons that control the legs and wings [17, 18]. Activation of the giant fibres initiates a motor response in which the fly jumps, thrusted by the mesothoracic legs, with no wing control and apparent directionality [19, 20]. Importantly, the probability of eliciting this jump response in wild-type red-eyed lines is reported to be relatively low (34–37%) in comparison with white-eyed lines (58–97%) [18].

In contrast, a visual looming stimulus that mimics approaching objects prompts a well-coordinated series of stereotypic movements as an escape response for both red-eyed and white-eyed flies [21]. These include preparatory leg movements for a directional motion followed by long or short take-offs, where wing extension depends on the required escape speed [12], similar to voluntary flight initiation [19]. Further dissection of the response to looming stimuli indicates that when take-off is not elicited, flies either exhibit running or freezing behaviours, where the probability of freezing is strongly dependent on baseline activity, as flies moving slower were more likely to freeze upon looming stimulation than flies moving faster [22].

However, most studies on visually evoked escape responses focus on the characterisation of the behavioural components within time frames of milliseconds to a few seconds. Thus, we employed DISCO to describe long-term changes in locomotion elicited by a single ‘lights-off’ stimulus presentation for both white-eyed and red-eyed flies. By using infrared-based detection of locomotion, we demonstrate a previously undescribed increase in activity elicited by a 1-s darkness presentation up to 30 s after stimuli. We show that this behaviour is present in white-eyed and red-eyed fly lines, although white-eyed flies are more sensitive to the stimuli. Moreover, we show that *Fmr1* null allele flies, a model of fragile X syndrome, do not exhibit the sudden darkness-induced increase in locomotion. We also show that the observed differences in locomotion after lights-off stimuli between fragile X and control flies cannot be accounted for by deficits in baseline locomotion, as the measured activity and speed before stimulation was comparable or higher to the control lines. Previous studies already reported deficits in odorant sensory responses in fragile X flies [23, 24]; however, to the best of our knowledge, this is the first description of deficits in visually evoked motor responses for this model.

Analysis of the response from single flies shows a highly variable response across individual flies and trials. Red-eyed CSORCs exhibited were completely unresponsive (delta values equal to zero) in 59% of stimuli presentations over the trials, while white-eyed *w*^*1118*^ flies failed to respond only 31% of the time. Interestingly, these ratios are similar to the previously reported jump response probability after lights-off [18]. Moreover, paired analyses of movement counts before and after stimuli showed that the behaviour of individual flies is significantly variable across trials, indicating that although the response is robust at a population level, further investigations are necessary to understand the factors driving the response of a single fly. By correlating locomotion values before and after stimuli, we also observed that flies with higher activity before lights-off were more prone to exhibit increased activity after stimuli, which resembles the literature reports using looming stimulation [22].

Additionally, by making use of the flexibility in the LED programming of the DISCO platform, we explored the different protocols of sudden darkness stimulation, showing that the locomotion response can be inhibited by repeated stimuli presentation. In white-eyed *w*^*1118*^ flies, this desensitisation occurred by repeated stimulation with intervals of 5 min, while for the red-eyed *CSORC* wild-type flies, the decrease in response only occurred by darkness presentation with 1-min intervals. Interestingly, when looking at individual trials, even initial ones, the average of delta movements is often reduced or even negative; however, after grouping averages in a bigger temporal frame (i.e. 30-min intervals), the group effect of increased mobility is more evident. We believe that due to the highly variable responses among individuals and trials, increasing the number of measurements by shortening the intra-trial intervals amplifies the visualisation of the variability of the results.

Taken together, our findings reinforce that *Drosophila* can respond and desensitise to sudden darkness (lights-off) presentation and confirm previous literature describing white-eyed flies as more sensitive to such stimuli. Nevertheless, these results should be interpreted with caution due to their relatively high variability at the level of individual flies and shorter inter-trial intervals, and further studies are needed to investigate the relationship between this behaviour and the widely studied jump response.

### Alcohol preference and circadian rhythm

The relationship between alcohol consumption and circadian rhythm has been widely studied in the literature, demonstrating that not only can ethanol intake alter the circadian clock and its mechanisms, but also a modulation of consumption depending on daytime [25]. For instance, human studies observed higher levels of alcohol consumption in individuals with evening chronotypes [26, 27]. Accordingly, alcohol intake also peaks at night hours in rodents, which naturally show a nocturnal profile of activity [28].

Due to similar circadian clock and reward systems, the fruit fly has also been a useful model to describe the interactions between the circadian clock and ethanol consumption [29]. Reports show that *Drosophila* exhibits higher sensitivity to alcohol during the mid to late-night phases, both under light-dark cycles and constant darkness [30, 31]. Recovery from the sedative effects was also significantly greater at night phases [30]. Notably, the circadian rhythmicity of sensitivity and tolerance to ethanol sedation is eliminated after mutation of certain circadian genes and under a constant light regime, corroborating the need for the circadian oscillator to modulate alcohol-induced sedation [30, 32]. However, these results were obtained by administering ethanol gas to the flies to assess their behaviour after consumption, while circadian patterns of active ethanol intake remained unexplored.

Thus, to investigate this question and demonstrate the use of DISCO for long-term experiments, we developed a 48-h ethanol preference test, where the lights were programmed into a 12:12 light-dark cycle. The interaction with the food options (with or without 15% ethanol) was inferred from the position of the flies measured by the infrared beams. We observed that ethanol preference happens intermittently throughout the day and was significantly higher in many hours of the dark phases. Correlation analysis of alcohol preference and daytime showed a small but significant trend for increased ethanol consumption during the dark phases. These results relate to results seen in rodents, which indicate higher alcohol consumption during the night [28]. They also implicate that flies tend to consume ethanol during periods of higher sensitivity to its effects. However, one should notice that the correlation analysis employed to test daytime trends in ethanol preference linearises the multimodal nature of the intake behaviour. Thus, further studies with bigger sample sizes are warranted to comprehensively describe if there are specific peaks of ethanol preference throughout the day. Additionally, future assays on DISCO could determine if specific circadian genes can influence the active seeking for ethanol intake, as for sensitivity and tolerance. Finally, although the indirect assessment of food interaction limits the interpretation of the data, these results are the first to indicate a role of light-dark phases on alcohol intake in flies.

### Light colour-based place preference

Place preference studies based on avoidance behaviours in *Drosophila* commonly rely on heat as an aversive stimulus, in which single freely walking flies are placed in a narrow box in complete darkness and conditioned to avoid one-half of the box by instantaneous heat presentation upon entering that area [33]. Alternatively, flies can be tested in a thermal–visual arena, where they can use environmental cues to find a hidden cool tile in an otherwise unappealing warm environment [34, 35]. However, the widespread use of assays based on a single type of aversive stimulus bring limitations when studying the machinery behind integrating different sensorial elements during learning.

With that in mind, we designed a protocol to explore the innate aversion of flies to blue light and their preference for green light [8]. The avoidance behaviour of blue light is shown to be independent of the circadian clock and relies on the TRPA channel *painless*, which is primary for nociception in flies, making this stimulus a good candidate for aversive-based behaviours [8]. Thus, we implemented a feedback loop protocol using DISCO, where the experimental tubes containing the flies were divided into two zones (i.e., left and right). When the flies crossed to a given zone, either blue or green LEDs were turned on.

As expected, flies learned to avoid the zones illuminated with blue light. Interestingly, *CSORC* flies show avoidance of the blue zones from the first hour of the training session, while *w*^*1118*^ flies display a slower learning curve. These results are in line with previous research showing that *w*^*1118*^ mutants perform worse than wild-type flies in heat-induced place conditioning tests, as they have lower levels of serotonin and dopamine [36]. To verify if this avoidance behaviour is in fact related to learning, *CSORC* flies were tested in a 10-h session, where the zones were interchanged at the 5th hour. We observed that after the zones were changed, the flies were unable to avoid the blue zones, indicating that the place preference is dependent on the association made in the initial hours of the session. Accordingly, it was shown that flies can integrate visual cues to learn place preference but changes in these cues disrupt learning [34]. Our results indicate that operant conditioning based on blue light exposure can be used as an additional tool for various studies, from sensory perception to learning and memory.

## Conclusions

Automated manipulation and analysis of behavioural tasks, such as those performed by DISCO, increase data precision, reliability, and experimental throughput. Thus, our method paves the way for a broader application and further protocol development of light-based assays for *Drosophila melanogaster*. The combination of these advances can bring immense benefits for the use of the fruit fly as a genetic model of cognitive and neurobiological impairments.

## Methods

### Fly strains and maintenance

For the experiments, male and female 5 to 7-day-old CSORC (originated from CantonS and OregonR-C lines), w1118, and fmr1 null-allele (fragile X syndrome model) strains of *Drosophila melanogaster* flies were used and kept at 25 °C, 12:12-h light/dark, 60% humidity. All lines were originated from Bloomington Drosophila Stock Center, Bloomington, IN, USA. Prior to experimentation, the flies were fed the Fisher-brand Jazz-Mix *Drosophila* food, complemented with 8.3% yeast extract (both from Fisher Scientific, Sweden).

### DISCO assembly and experimentation

The DISCO apparatus is based on the MB5 MultiBeam Activity Monitor (TriKinetics Inc., USA), which has 16 independent tube slots, each with 17 infrared beams (IR) for movement assessment. Two devices were used, allowing for testing 32 flies at a time. Stripes of RGB LED lights were connected to the MB5 Monitor behind the IR beams (Fig. 1). The lights are regulated by an Arduino Uno microcontroller (Arduino, Italy), which in turn is programmed to communicate with the MATLAB environment version R2018b (MathWorks, USA) through the Legacy NeoPixel Add-On Library. The measured light intensity was 0.3 W/cm^2^. Both the Arduino code for MATLAB communication and the MATLAB codes custom-made for each experimental protocol described in the ‘Results’ section can be found as additional material.

### Darkness-induced locomotion analysis

**Fig. 6:**
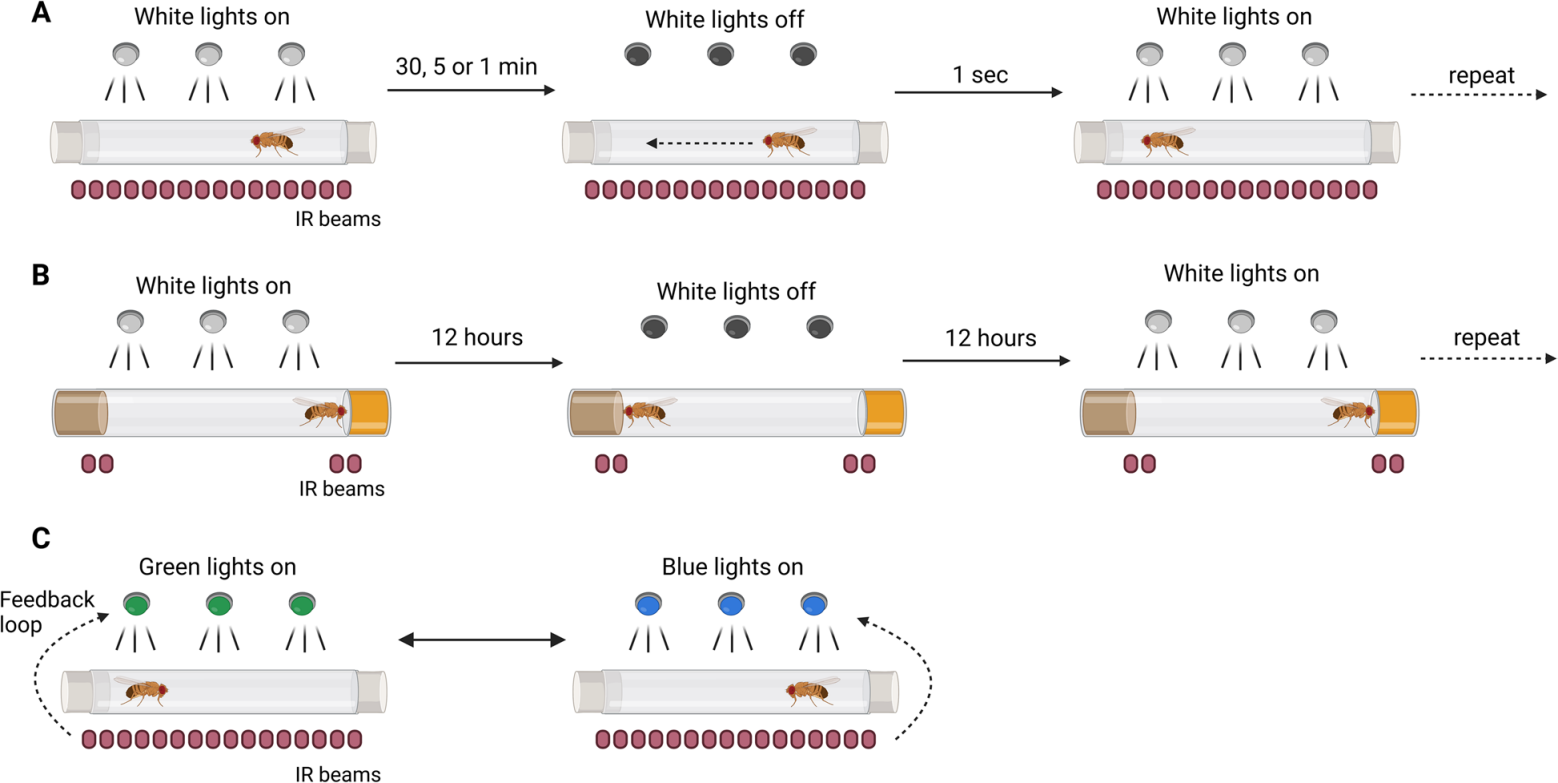
Illustration of protocols used in the study. **A** Protocol for darkness-induced locomotion assessment. **B** Protocol for the alcohol preference assay. **C** Protocol for the feedback loop place preference test

To perform the darkness-induced locomotion experiments, flies underwent CO_2_ anaesthesia and were individually placed in glass tubes sealed with fly food, cotton buds, and a lid 24 h prior to the task. The tubes were horizontally positioned in the apparatus (Fig. 1), which constantly recorded the location and movement of the flies. The experiments were taken place between 10 a.m. and 11 a.m. (ZT 2–3). After a habituation period of 30 min, the automatically controlled light stimulation started (Fig. 6A). Once all stimulation sessions were completed, the raw data from the activity monitor device was analysed by custom MATLAB routines. The number of beam crossings (named ‘counts’) was measured for each second (the shortest resolution time) by the MB5 MultiBeam Activity Monitor, used in the literature to compute locomotion from larvae and adult flies [37, 38]. Simultaneously, the exact moments of darkness stimuli presentation were recorded by the MATLAB-Arduino system of light control in an independent text file. The raw output data from the MB5 monitor was then converted to CSV files using the DamFileScan software from TriKinetics.

The delta response index was calculated by a custom-made MATLAB routine, which reads the activity measure file and the ‘sudden darkness’ presentation times, by subtracting the average movement counts for 30 s after a given startle stimulus from the measure for 30 s before the same point. The exact second in which the stimulus was presented was ignored. This index allows the visualisation of the increase (or decrease) in movement, taking into consideration the baseline movement just before the stimuli. Flies that showed no activity before and after stimuli through all sessions were considered dead and thus excluded from the analysis.

### Long-term alcohol preference assay

For the alcohol preference experiments, flies were anaesthetised with CO_2_ and individually placed in glass tubes sealed with low-sugar food, alcoholic food, and cotton buds. The low-sugar food was composed of 5% sucrose, 5% yeast, and 2% agarose, while the alcoholic food was made with the same recipe adding 15% ethanol. To avoid possible confounders due to CO_2_ anaesthesia, the data was analysed circa 12 h after the placement in the tubes, starting at ZT 0 (8 a.m.). DISCO was programmed for a 12:12 light/dark cycle, and the position of the flies was recorded for 48 h. Food preference was inferred by the percentage of time per hour spent at the tube ends (Fig. 6B), after analysis of the raw data by a custom MATLAB routine.

### Feedback-loop place preference assay

For the place preference test, flies underwent CO_2_ anaesthesia and were individually placed in glass tubes sealed with cotton buds and a lid 3 to 4 h before experimentation. The experiments were taken place between 11 a.m. and 12 a.m. (ZT 3–4) At the beginning of the test, the tube was divided into two zones (i.e. left and right zones), where they were assigned either blue or green illumination. A custom-made MATLAB code was made to control the LEDs while reading the position output from the MB5 infra-red equipment. Once a fly crossed into a given zone, all LEDs corresponding to the fly were lighted in the respective colour (Fig. 6C). The colour preference of the flies was calculated by the percentage of time per hour spent in either blue or green zones.

### Statistical analyses

All analyses were performed with the GraphPrism 8 software. For experiments testing the effect of a given intervention on different groups measured over time (or trials) (Figs. 2B and 4A, B; Fig. S1), we employed a two-way repeated measures ANOVA. When the intervention effect was measured over time, but in the same group of flies in different conditions (i.e., place preference, Fig. 5A, B; before-after stimuli, Additional file 2: Fig. S2 B-D), the two-way ANOVA with repeated measures by both factors was used. Simple comparisons of group averages were performed with one-way ANOVA (Figs. 2C and 3A, B; Additional file 4: Fig. S4 A-B). ANOVAs were followed by Holm-Sidak post hoc tests for comparisons among individual pairs of groups (Fig. 2B, C; Additional file 4: Fig. S4 A-B), Fisher’s LSD post hoc test for individual time point comparisons (Fig. 4A, B), and linear-trend post hoc test, which assesses for linear increment or reduction across groups averages, for effect changes over time in individual groups (Fig. 3A, B). The latter test was implemented using the GraphPad Prism software, and more details on the method can be found on the software’s website (https://www.graphpad.com/support/faq/the-post-test-for-trend). Lastly, Pearson’s correlation test was used in Fig. 4C, D and Additional file 3: Fig. S3. The statistical tests used for each experiment and the resulting *p*-values are also described in the legend of their respective figures. Additionally, we added all statistical results and tables as supplementary material for consultation.

## Supplementary Information

**Additional file 1: Figure S1.** Effects of transient darkness on distinct sexes. Delta locomotion response index for males and females from CSORC and w1118 lines during the 6 trials. No significant differences were found between groups when dividing flies by sex (*p*=0.218 and *p*=0.652 for sex differences for CSORC and w1118 lines, respectively; two-way ANOVA).

**Additional file 2: Figure S2.** Analysis of darkness-induced locomotion by individual flies. A. Values of delta movement index for each trial, as shown in Figure 1, with single values plotted. B. Paired movement counts before and after stimuli for CSORC flies (p_(before-after)_=0.0002, p_(trials)_=0.0419); C. for w1118 flies (p_(before-after)_=0.0001, p_(trials)_=0.0429); D. for Fragile X flies (p_(before-after)_=0.9303, p_(trials)_=0.0489). Analyses in B-D were performed using two-way ANOVA, repeated measures by both factors.

**Additional file 3: Figure S3.** Correlation of movement counts before and after stimuli. The flies’ movements measured preceding and posterior to sudden darkness presentation are statistically correlated for A. CSORC (*r*=0.501; *p*<0.0001), B. w1118 (*r*=0.559; *p*<0.0001), and C. Fragile X lines (*r*=0.476; *p*<0.0001).

**Additional file 4: Figure S4.** Baseline activity and speed. A CSORC flies were significantly less active than w1118 (*p*=0.0005) and Fragile X flies (*p*=0.001), while w1118 and Fragile X groups were comparable (*p*=0.727). B. average baseline speed of Fragile X flies was significantly greater than CSORCs (*p*=0.005), but smaller than w1118 controls (*p*=0.005). One-way ANOVA was used in both comparisons.

**Additional file 5: Video S1.** Increased locomotion after lights-off stimuli presentation.

**Additional file 6.** Codes and Data. MATLAB codes and GraphPad Prism files.

**Additional file 7.** Statistical tables. Complete description of statistical results.

## Data Availability

All data generated or analysed during this study are included in this published article and its supplementary information files. We made available the MATLAB codes necessary for the implementation of the Arduino-controlled LEDs in DISCO, as well as all data and statistical analyses described in the figures (as GraphPad Prism files).

## Abbreviations

DISCO: Drosophila Interactive System for Controlled Optical manipulations
LED: Light-emitting diode
FMR1: Fragile X messenger ribonucleoprotein 1
ANOVA: Analysis of variance statistical test
IR: Infra-red
