# Supplementary figures and images for: A simple high-throughput method for automated detection of Drosophila melanogaster light-dependent behaviours
Source: BMC Biol. 2022 Dec 17;20:283. doi: 10.1186/s12915-022-01476-z (PMC9758938; doi:10.1186/s12915-022-01476-z)

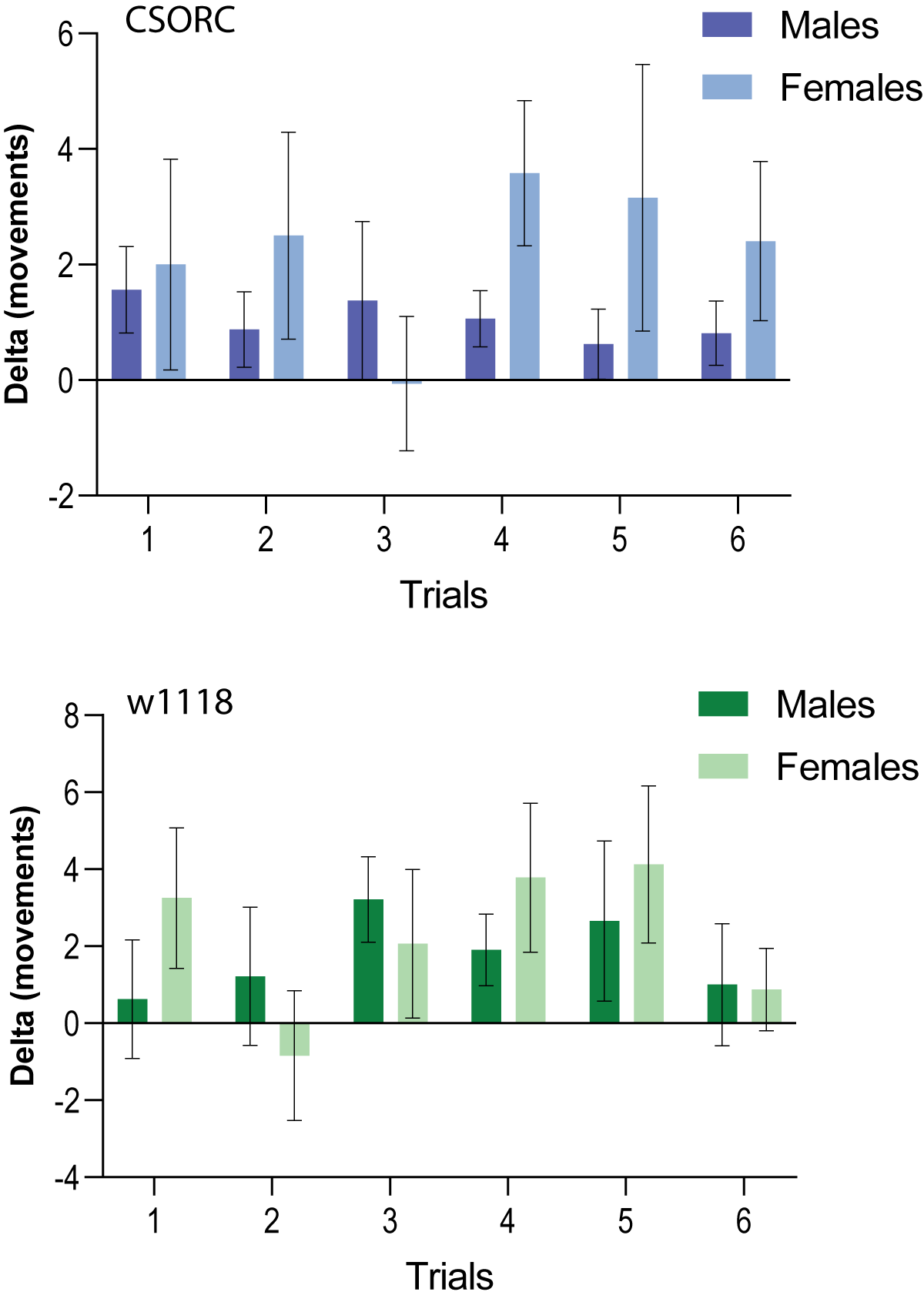

Supplement: Supplementary file 1 — Additional file 1: Figure S1. Effects of transient darkness on distinct sexes. Delta locomotion response index for males and females from CSORC and w1118 lines during the 6 trials. No significant differences were found between groups when dividing flies by sex (p=0.218 and p=0.652 for sex differences for CSORC and w1118 lines, respectively; two-way ANOVA). [file 12915_2022_1476_MOESM1_ESM.tif]

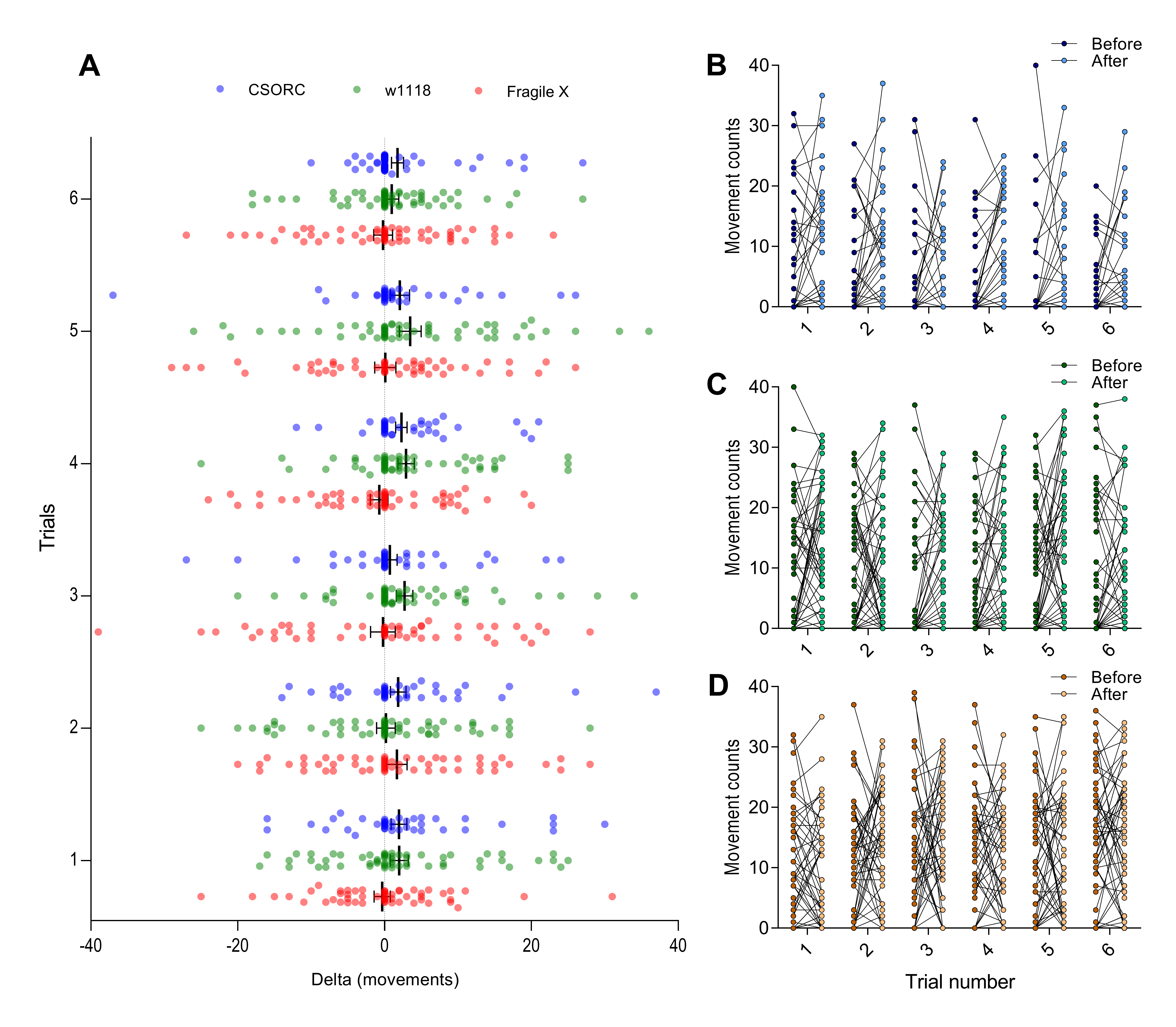

Supplement: Supplementary file 2 — Additional file 2: Figure S2. Analysis of darkness-induced locomotion by individual flies. A. Values of delta movement index for each trial, as shown in Figure 1, with single values plotted. B. Paired movement counts before and after stimuli for CSORC flies (p(before-after)=0.0002, p(trials)=0.0419); C. for w1118 flies (p(before-after)=0.0001, p(trials)=0.0429); D. for Fragile X flies (p(before-after)=0.9303, p(trials)=0.0489). Analyses in B-D were performed using two-way ANOVA, repeated measures by both factors. [file 12915_2022_1476_MOESM2_ESM.tif]

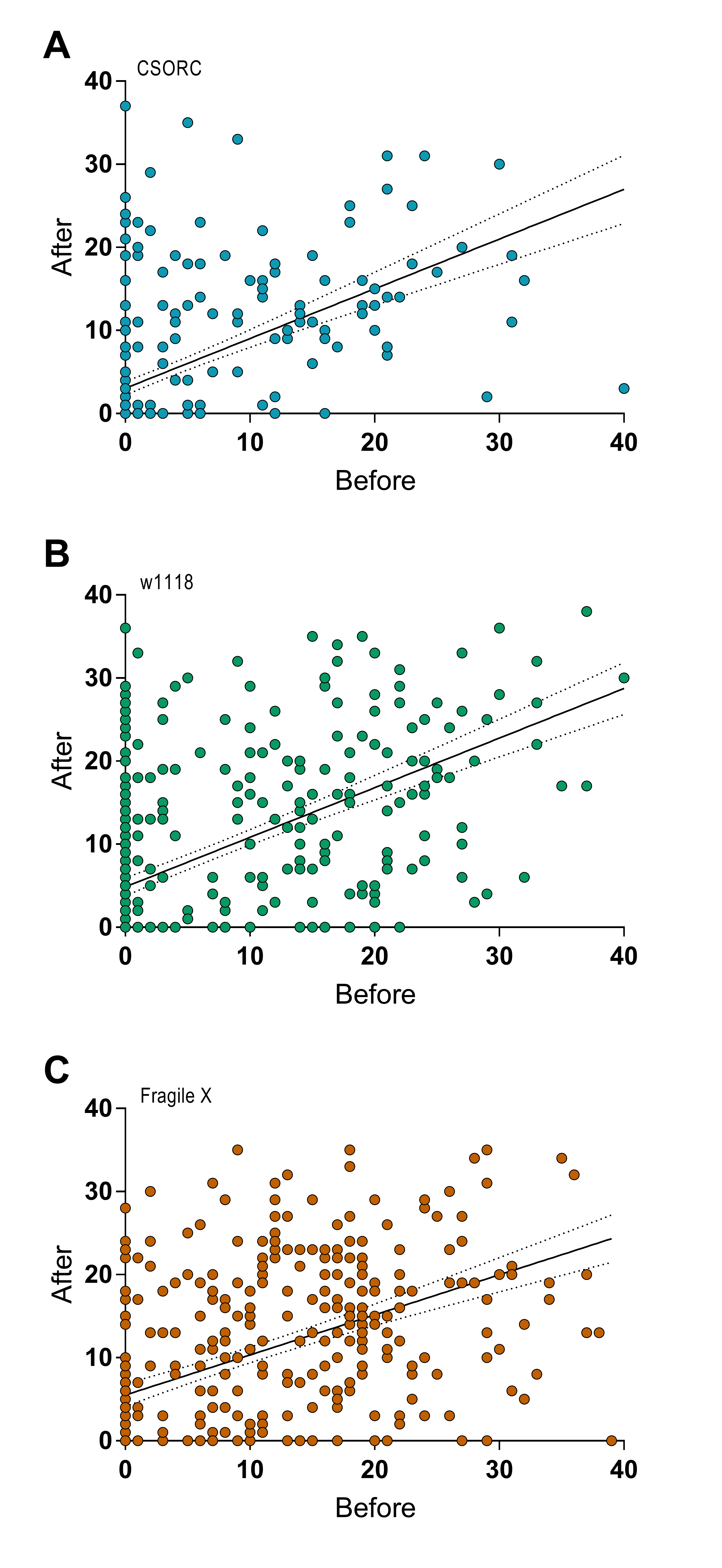

Supplement: Supplementary file 3 — Additional file 3: Figure S3. Correlation of movement counts before and after stimuli. The flies’ movements measured preceding and posterior to sudden darkness presentation are statistically correlated for A. CSORC (r=0.501; p<0.0001), B. w1118 (r=0.559; p<0.0001), and C. Fragile X lines (r=0.476; p<0.0001). [file 12915_2022_1476_MOESM3_ESM.tif]

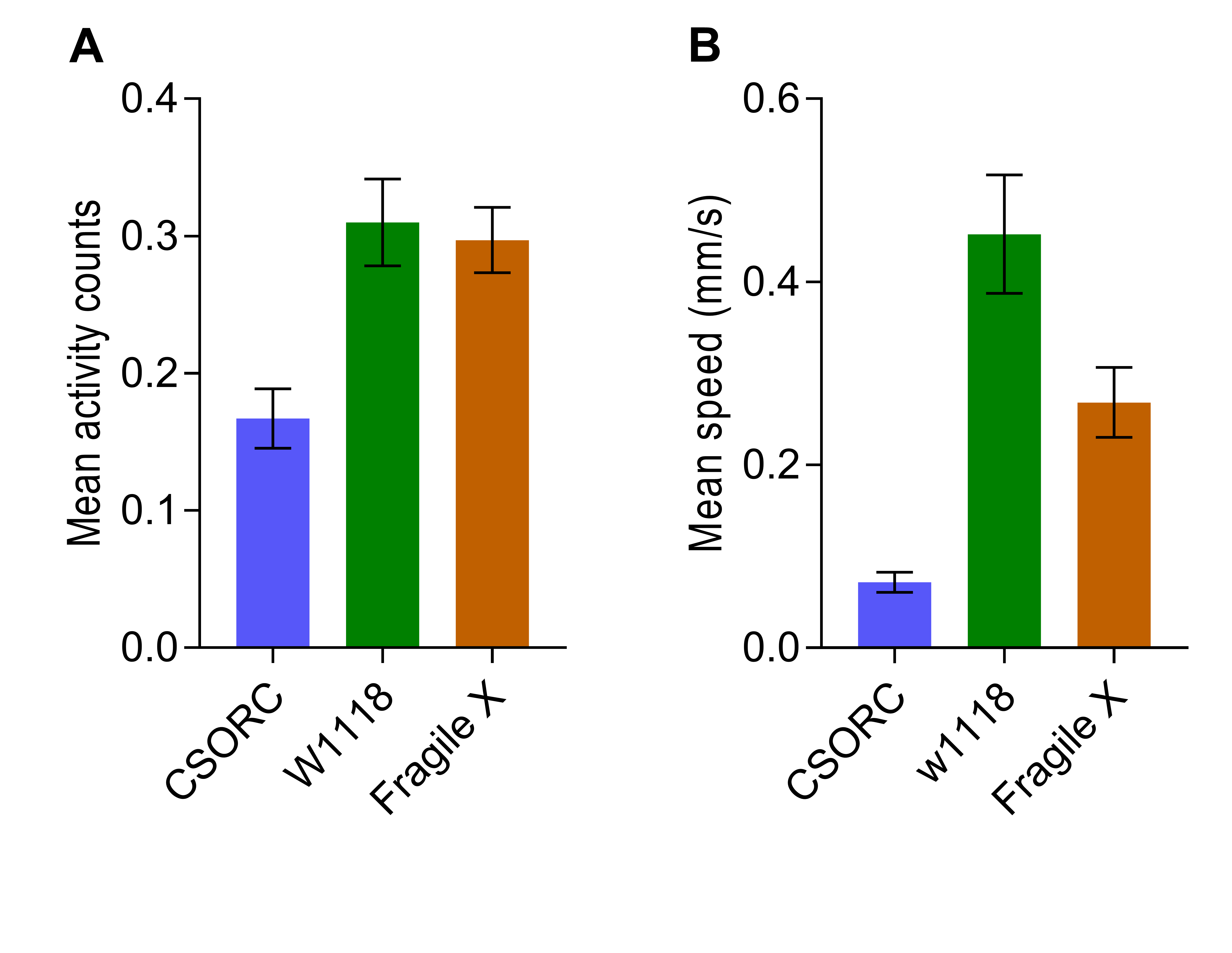

Supplement: Supplementary file 4 — Additional file 4: Figure S4. Baseline activity and speed. A CSORC flies were significantly less active than w1118 (p=0.0005) and Fragile X flies (p=0.001), while w1118 and Fragile X groups were comparable (p=0.727). B. average baseline speed of Fragile X flies was significantly greater than CSORCs (p=0.005), but smaller than w1118 controls (p=0.005). One-way ANOVA was used in both comparisons. [file 12915_2022_1476_MOESM4_ESM.tif]

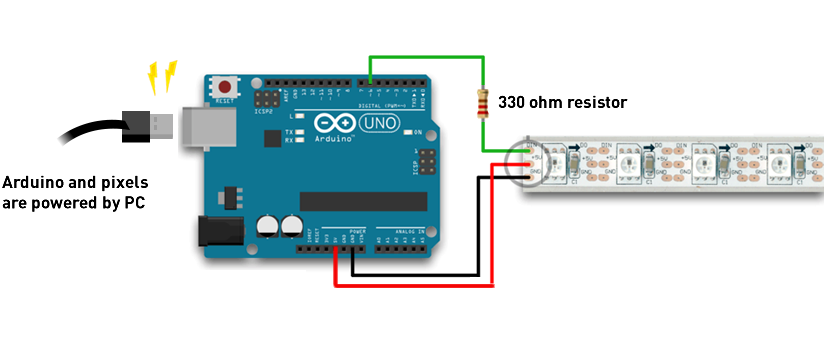

Supplement: Supplementary file 6 — Additional file 6. Codes and Data. MATLAB codes and GraphPad Prism files. [file 12915_2022_1476_MOESM6_ESM.zip › Supplementary Material/DISCO codes/Arduino assembly.png]
