# Supplementary material for: A simple high-throughput method for automated detection of Drosophila melanogaster light-dependent behaviours
Source: BMC Biol. 2022 Dec 17;20:283. doi: 10.1186/s12915-022-01476-z (PMC9758938; doi:10.1186/s12915-022-01476-z)
Supplement: Supplementary file 7 — Additional file 7. Statistical tables. Complete description of statistical results. [file 12915_2022_1476_MOESM7_ESM.pdf]

Figure 2B – Two-way Repeated Measures ANOVA (Trials as repeated measures)

Source of Variation	% of total variation	p value	p value summary	Significant?
**Interaction**	0,7138	0,6587	ns	No
**Trial**	0,1277	0,9151	ns	No
**Line**	0,9057	0,0108	*	Yes
**Subject**	17,05	0,3277	ns	No

ANOVA table	SS	DF	MS	F (DFn, DFd)	p value
**Interaction**	660,1	10	66,01	F (10, 875) = 0,7693	P=0,6587
**Trial**	118,1	5	23,63	F (4,595, 804,1) = 0,2753	P=0,9151
**Line**	837,6	2	418,8	F (2, 175) = 4,647	P=0,0108
**Subject**	15771	175	90,12	F (175, 875) = 1,050	P=0,3277
**Residual**	75085	875	85,81		

Holm-Sidak's multiple comparisons	Mean Diff,	Significant?	Summary	Adjusted p Value
**CSORC vs. w1118**	-0,2693	No	ns	0,6768
**CSORC vs. Fragile X**	1,736	Yes	*	0,0245
**w1118 vs. Fragile X**	2,005	Yes	*	0,0213

Test details	Mean 1	Mean 2	Mean Diff	SE of Diff	N1	N2	t	DF
**CSORC vs. w1118**	1,784	2,054	-0,2693	0,6458	348	372	0,4170	703,2
**CSORC vs. Fragile X**	1,784	0,04885	1,736	0,6914	348	348	2,510	642,5
**w1118 vs. Fragile X**	2,054	0,04885	2,005	0,7432	372	348	2,698	703,4

Figure 2C – One-way ANOVA

ANOVA summary	
**F**	4,647
**P value**	0,0108
**P value summary**	*
**Significant diff. among means (P < 0.05)?**	Yes
**R square**	0,05043

ANOVA table	SS	DF	MS	F (DFn, DFd)	p value
**Treatment (between columns)**	139,6	2	69,8	F (2, 175) = 4,647	P=0,0108
**Residual (within columns)**	2628	175	15,02		
**Total**	2768	177			

Holm-Sidak's multiple comparisons	Mean Diff	Significant?	Summary	Adjusted p value
**CSORC vs. w1118**	-0,2693	No	ns	0,7041
**CSORC vs. Fragile X**	1,736	Yes	*	0,0335
**w1118 vs. Fragile X**	2,005	Yes	*	0,0154

Test details	Mean 1	Mean 2	Mean Diff	SE of diff	n1	n2	t	DF
**CSORC vs. w1118**	1,784	2,054	-0,2693	0,708	58	62	0,3804	175
**CSORC vs. Fragile X**	1,784	0,04885	1,736	0,7197	58	58	2,412	175
**w1118 vs. Fragile X**	2,054	0,04885	2,005	0,708	62	58	2,832	175

Figure 3A (5 min ITI) – One-way ANOVA

ANOVA summary	
**F**	0,4326
**p value**	0,7298
**p value summary**	ns
**Significant diff. among means (P < 0.05)?**	No
**R square**	0,003404

ANOVA table	SS	DF	MS	F (DFn, DFd)	p value
**Treatment (between columns)**	13,8	3	4,602	F (3, 380) = 0,4326	P=0,7298
**Residual (within columns)**	4042	380	10,64		
**Total**	4056	383			

Post test for linear trend	
**Slope**	0,04167
**p value**	0,7797
**p value summary**	ns
**Is linear trend significant (P < 0.05)?**	No

ANOVA table	SS	DF	MS	R square (alerting)	R square (effect size)	F (DFn, DFd)	P value
**Treatment (between columns)**	13,8	3	4,602				
**Linear**	0,8333	1	0,8333	0,06036	0,0002055	F (1, 380) = 0,0783	P=0,7797
**Nonlinear**	12,97	2	6,486	0,9396	0,003198	F (2, 380) = 0,6098	P=0,5440
**Residual (within columns)**	4042	380	10,64				
**Total**	4056	383					

Figure 3A (1 min ITI) – One-way ANOVA

ANOVA summary	
**F**	4,693
**p value**	0,0031
**p value summary**	**
**Significant diff. among means (P < 0.05)?**	Yes
**R square**	0,03573

ANOVA table	SS	DF	MS	F (DFn, DFd)	p value
**Treatment (between columns)**	12	3	4	F (3, 380) = 4,693	P=0,0031
**Residual (within columns)**	323,9	380	0,8523		
**Total**	335,9	383			

Post test for linear trend	
**Slope**	-0,1526
**p value**	0,0003
**p value summary**	***
**Is linear trend significant (P < 0.05)?**	Yes

ANOVA table	SS	DF	MS	R square (alerting)	R square (effect size)	F (DFn, DFd)	P value
**Treatment (between columns)**	12	3	4				
**Linear**	11,18	1	11,18	0,9318	0,03329	F (1, 380) = 13,12	P=0,0003
**Nonlinear**	0,8179	2	0,4089	0,06816	0,002435	F (2, 380) = 0,4798	P=0,6193
**Residual (within columns)**	323,9	380	0,8523				
**Total**	335,9	383					

Figure 3B (5 min ITI) – One-way ANOVA

ANOVA summary	
**F**	2,071
**p value**	0,1036
**p value summary**	ns
**Significant diff. among means (P < 0.05)?**	No
**R square**	0,01608

ANOVA table	SS	DF	MS	F (DFn, DFd)	p value
**Treatment (between columns)**	67,08	3	22,36	F (3, 380) = 2,071	P=0,1036
**Residual (within columns)**	4103	380	10,8		
**Total**	4170	383			

Post test for linear trend	
**Slope**	-0,3069
**p value**	0,0414
**p value summary**	*
**Is linear trend significant (P < 0.05)?**	Yes

ANOVA table	SS	DF	MS	R square (alerting)	R square (effect size)	F (DFn, DFd)	P value
**Treatment (between columns)**	67,08	3	22,36				
**Linear**	45,22	1	45,22	0,6742	0,01084	F (1, 380) = 4,188	P=0,0414
**Nonlinear**	21,85	2	10,93	0,3258	0,00524	F (2, 380) = 1,012	P=0,3645
**Residual (within columns)**	4103	380	10,8				
**Total**	4170	383					

Figure 3B (1 min ITI) – One-way ANOVA

ANOVA summary	
**F**	0,4047
**p value**	0,7497
**p value summary**	ns
**Significant diff. among means (P < 0.05)?**	No
**R square**	0,003184

ANOVA table	SS	DF	MS	F (DFn, DFd)	p value
**Treatment (between columns)**	2,192	3	0,7307	F (3, 380) = 0,4047	P=0,7497
**Residual (within columns)**	686,1	380	1,806		
**Total**	688,3	383			

Post test for linear trend	
**Slope**	0,03926
**p value**	0,5225
**p value summary**	ns
**Is linear trend significant (P < 0.05)?**	No

ANOVA table	SS	DF	MS	R square (alerting)	R square (effect size)	F (DFn, DFd)	P value
**Treatment (between columns)**	2,192	3	0,7307				
**Linear**	0,7397	1	0,7397	0,3375	0,001075	F (1, 380) = 0,4097	P=0,5225
**Nonlinear**	1,452	2	0,7261	0,6625	0,00211	F (2, 380) = 0,4022	P=0,6692
**Residual (within columns)**	686,1	380	1,806				
**Total**	688,3	383					

Figure 4A – Two-way Repeated Measures ANOVA (Time as repeated measures)

Source of Variation	% of total variation	p value	p value summary	Significant?
**Interaction**	3,000	0,1137	ns	No
**Time**	11,99	<0,0001	****	Yes
**Food**	1,866	0,0860	ns	No
**Subject**	16,50	<0,0001	****	Yes

ANOVA table	SS	DF	MS	F (DFn, DFd)	p value
**Interaction**	20161	47	429,0	F (47, 1316) = 1,260	P=0,1137
**Time**	80546	47	1714	F (9,027, 252,7) = 5,035	P<0,0001
**Food**	12543	1	12543	F (1, 28) = 3,167	P=0,0860
**Subject**	110896	28	3961	F (28, 1316) = 11,64	P<0,0001
**Residual**	447881	1316	340,3		

Uncorrected Fisher's LSD	Mean Diff,	95,00% CI of diff	Below threshold?	Summary	Individual p value
**Alcohol vs Normal food (ZT)**					
**1**	0,2709	-10,30 to 10,84	No	ns	0,9584
**2**	-3,555	-12,98 to 5,867	No	ns	0,4369
**3**	5,021	-2,058 to 12,10	No	ns	0,1520
**4**	-5,653	-20,26 to 8,955	No	ns	0,4303
**5**	-11,40	-30,86 to 8,073	No	ns	0,2304
**6**	-7,718	-22,07 to 6,639	No	ns	0,2684
**7**	-2,126	-16,76 to 12,51	No	ns	0,7653
**8**	3,122	-16,01 to 22,25	No	ns	0,7406
**9**	6,927	-9,836 to 23,69	No	ns	0,3998
**10**	10,55	-3,970 to 25,07	No	ns	0,1422
**11**	15,41	0,04387 to 30,78	Yes	*	0,0494
**12**	2,496	-4,789 to 9,781	No	ns	0,4886
**13**	14,60	4,087 to 25,12	Yes	**	0,0086
**14**	3,978	-9,715 to 17,67	No	ns	0,5566
**15**	2,303	-6,034 to 10,64	No	ns	0,5744
**16**	5,214	-5,046 to 15,47	No	ns	0,3029
**17**	8,613	-0,2936 to 17,52	No	ns	0,0572
**18**	-8,337	-21,80 to 5,129	No	ns	0,2086
**19**	4,029	-4,517 to 12,57	No	ns	0,3384

**20**	3,932	-7,282 to 15,15	No	ns	0,4786
**21**	0,03058	-4,267 to 4,328	No	ns	0,9885
**22**	2,815	-3,058 to 8,688	No	ns	0,3320
**23**	7,704	0,6086 to 14,80	Yes	*	0,0347
**24**	9,442	-0,1401 to 19,02	No	ns	0,0532
**1**	19,05	0,2842 to 37,82	Yes	*	0,0469
**2**	24,44	3,739 to 45,14	Yes	*	0,0232
**3**	19,65	-2,113 to 41,41	No	ns	0,0740
**4**	15,27	-5,858 to 36,39	No	ns	0,1470
**5**	6,214	-13,26 to 25,69	No	ns	0,5128
**6**	8,308	-10,57 to 27,19	No	ns	0,3642
**7**	1,140	-20,33 to 22,61	No	ns	0,9141
**8**	-0,6569	-22,57 to 21,26	No	ns	0,9514
**9**	10,34	-13,77 to 34,45	No	ns	0,3864
**10**	4,675	-16,80 to 26,15	No	ns	0,6589
**11**	8,555	-3,177 to 20,29	No	ns	0,1452
**12**	11,35	0,05043 to 22,66	Yes	*	0,0491
**13**	11,07	-0,3432 to 22,47	No	ns	0,0568
**14**	13,01	-0,01087 to 26,04	No	ns	0,0502
**15**	1,872	-16,28 to 20,03	No	ns	0,8343
**16**	-6,807	-23,62 to 10,00	No	ns	0,4138
**17**	3,868	-17,21 to 24,95	No	ns	0,7099
**18**	7,049	-9,158 to 23,26	No	ns	0,3804
**19**	14,65	1,911 to 27,39	Yes	*	0,0264
**20**	10,76	-2,216 to 23,73	No	ns	0,0995
**21**	0,9929	-16,30 to 18,29	No	ns	0,9072
**22**	8,482	-8,879 to 25,84	No	ns	0,3219
**23**	13,55	-4,007 to 31,10	No	ns	0,1234
**24**	8,823	-10,82 to 28,47	No	ns	0,3619

Figure 4B – Two-way Repeated Measures ANOVA (Time as repeated measures)

Source of Variation	% of total variation	p value	p value summary	Significant?
**Interaction**	3,346	0,0396	*	Yes
**Time**	11,41	<0,0001	****	Yes
**Food**	1,005	0,2874	ns	No
**Subject**	22,16	<0,0001	****	Yes

ANOVA table	SS	DF	MS	F (DFn, DFd)	p value
**Interaction**	19704	47	419,2	F (47, 1222) = 1,401	P=0,0396
**Time**	67191	47	1430	F (7,428, 193,1) = 4,779	P<0,0001
**Food**	5920	1	5920	F (1, 26) = 1,180	P=0,2874
**Subject**	130464	26	5018	F (26, 1222) = 16,77	P<0,0001
**Residual**	365588	1222	299,2		

Uncorrected Fisher's LSD	Mean Diff,	95,00% CI of diff	Below threshold?	Summary	Individual p value
**Alcohol vs Normal food (ZT)**					
**1**	-8,271	-18,50 to 1,955	No	ns	0,1067
**2**	-2,980	-12,09 to 6,128	No	ns	0,5053
**3**	-2,275	-11,36 to 6,815	No	ns	0,6109
**4**	-4,994	-14,23 to 4,245	No	ns	0,2724
**5**	-4,594	-20,19 to 11,00	No	ns	0,5439
**6**	-3,412	-18,55 to 11,73	No	ns	0,6423
**7**	6,290	-8,629 to 21,21	No	ns	0,3912
**8**	8,348	-3,646 to 20,34	No	ns	0,1590
**9**	0,4896	-10,17 to 11,15	No	ns	0,9255
**10**	-2,588	-16,23 to 11,05	No	ns	0,6976
**11**	3,869	-6,045 to 13,78	No	ns	0,4297
**12**	5,618	-10,09 to 21,32	No	ns	0,4669
**13**	21,73	6,255 to 37,21	Yes	**	0,0087
**14**	-4,457	-24,69 to 15,78	No	ns	0,6544
**15**	-1,100	-21,49 to 19,29	No	ns	0,9125
**16**	-4,771	-19,31 to 9,768	No	ns	0,5035
**17**	1,939	-8,092 to 11,97	No	ns	0,6941
**18**	2,350	-13,33 to 18,03	No	ns	0,7580
**19**	5,576	-7,568 to 18,72	No	ns	0,3827

**20**	5,104	-10,78 to 20,99	No	ns	0,5085
**21**	9,309	-5,525 to 24,14	No	ns	0,2019
**22**	-7,326	-20,77 to 6,117	No	ns	0,2722
**23**	-1,285	-12,66 to 10,09	No	ns	0,8172
**24**	6,936	-12,52 to 26,39	No	ns	0,4681
**1**	12,33	-6,930 to 31,59	No	ns	0,1942
**2**	12,06	-3,858 to 27,98	No	ns	0,1293
**3**	1,519	-10,91 to 13,95	No	ns	0,8031
**4**	0,1584	-19,81 to 20,13	No	ns	0,9871
**5**	-3,652	-20,24 to 12,94	No	ns	0,6532
**6**	-1,001	-17,83 to 15,82	No	ns	0,9036
**7**	4,031	-13,19 to 21,25	No	ns	0,6314
**8**	4,847	-12,58 to 22,27	No	ns	0,5724
**9**	0,2785	-10,79 to 11,35	No	ns	0,9591
**10**	4,565	-9,706 to 18,84	No	ns	0,5146
**11**	-0,3649	-13,80 to 13,07	No	ns	0,9558
**12**	8,723	-2,589 to 20,03	No	ns	0,1241
**13**	3,092	-9,042 to 15,23	No	ns	0,6037
**14**	0,4110	-14,36 to 15,18	No	ns	0,9548
**15**	3,846	-10,95 to 18,64	No	ns	0,5950
**16**	8,789	-10,79 to 28,36	No	ns	0,3637
**17**	22,06	2,994 to 41,13	Yes	*	0,0259
**18**	18,49	-1,682 to 38,66	No	ns	0,0701
**19**	16,61	-2,708 to 35,94	No	ns	0,0874
**20**	11,63	-8,219 to 31,47	No	ns	0,2390
**21**	16,93	-2,197 to 36,06	No	ns	0,0796
**22**	19,64	1,887 to 37,40	Yes	*	0,0317
**23**	10,40	-9,472 to 30,26	No	ns	0,2886
**24**	-3,429	-23,90 to 17,05	No	ns	0,7333

Figure 4C – Pearson’s Correlation

Pearson r	
**r**	0,07731
**95% confidence interval**	0,004268 to 0,1495
**R squared**	0,005977

P value	
**P (two-tailed)**	0,0381
**P value summary**	*
**Significant? (alpha = 0.05)**	Yes
**Number of XY Pairs**	720

Figure 4D – Pearson’s Correlation

Pearson r	
**r**	0,2016
**95% confidence interval**	0,1279 to 0,2731
**R squared**	0,04064

P value	
**P (two-tailed)**	<0,0001
**P value summary**	****
**Significant? (alpha = 0.05)**	Yes
**Number of XY Pairs**	672

Figure 5A (males) – Two-way Repeated Measures ANOVA (repeated measures by both factors)

Source of Variation	% of total variation	P value	P value summary	Significant?
**Time**	0,005077	0,4174	ns	No
**Place**	12,33	<0,0001	****	Yes
**Interaction: Time x Place**	1,393	0,0377	*	Yes
**Interaction: Time x Subjects**	0,4011			
**Interaction: Place x Subjects**	39,72			
**Subjects**	0,01173			

ANOVA table	SS	DF	MS	F (DFn, DFd)	P value
**Time**	17,23	5	3,447	F (5, 395) = 1	P=0,4174
**Place**	41860	1	41860	F (1, 79) = 24,52	P<0,0001
**Interaction: Time x Place**	4728	5	945,6	F (5, 395) = 2,385	P=0,0377
**Interaction: Time x Subjects**	1362	395	3,447		
**Interaction: Place x Subjects**	134842	79	1707		
**Subjects**	39,83	79	0,5042		
**Residual**	156616	395	396,5		

Figure 5A (females) – Two-way Repeated Measures ANOVA (repeated measures by both factors)

Source of Variation	% of total variation	P value	P value summary	Significant?
**Time**	1,527e-029	<0,0001	****	Yes
**Place**	22,47	0,0005	***	Yes
**Interaction: Time x Place**	1,113	0,4103	ns	No
**Interaction: Time x Subjects**	3,952e-029			
**Interaction: Place x Subjects**	43,56			
**Subjects**	2,956e-030			

ANOVA table	SS	DF	MS	F (DFn, DFd)	P value
**Time**	2,817e-026	5	5,634e-027	F (5, 150) = 11,59	P<0,0001
**Place**	41457	1	41457	F (1, 30) = 15,48	P=0,0005
**Interaction: Time x Place**	2052	5	410,5	F (5, 150) = 1,016	P=0,4103
**Interaction: Time x Subjects**	7,29e-026	150	4,86e-028		
**Interaction: Place x Subjects**	80360	30	2679		
**Subjects**	5,453e-027	30	1,818e-028		
**Residual**	60591	150	403,9		

Figure 5B (males) – Two-way Repeated Measures ANOVA (repeated measures by both factors)

Source of Variation	% of total variation	P value	P value summary	Significant?
**Time**	1,448e-028	<0,0001	****	Yes
**Place**	18,41	<0,0001	****	Yes
**Interaction: Time x Place**	3,374	0,0014	**	Yes
**Interaction: Time x Subjects**	1,762e-028			
**Interaction: Place x Subjects**	39,34			
**Subjects**	3,206e-029			

ANOVA table	SS	DF	MS	F (DFn, DFd)	P value
**Time**	3,393e-025	5	6,785e-026	F (5, 235) = 38,61	P<0,0001
**Place**	43144	1	43144	F (1, 47) = 22	P<0,0001
**Interaction: Time x Place**	7907	5	1581	F (5, 235) = 4,079	P=0,0014
**Interaction: Time x Subjects**	4,13e-025	235	1,757e-027		
**Interaction: Place x Subjects**	92191	47	1962		
**Subjects**	7,512e-026	47	1,598e-027		
**Residual**	91115	235	387,7		

Figure 5B (females) – Two-way Repeated Measures ANOVA (repeated measures by both factors)

Source of Variation	% of total variation	P value	P value summary	Significant?
**Time**	5,31e-028	<0,0001	****	Yes
**Place**	13,33	<0,0001	****	Yes
**Interaction: Time x Place**	4,197	0,0003	***	Yes
**Interaction: Time x Subjects**	5,294e-028			
**Interaction: Place x Subjects**	27,26			
**Subjects**	2,418e-028			

ANOVA table	SS	DF	MS	F (DFn, DFd)	P value
**Time**	1,047e-024	5	2,094e-025	F (5, 315) = 63,2	P<0,0001
**Place**	26277	1	26277	F (1, 63) = 30,8	P<0,0001
**Interaction: Time x Place**	8275	5	1655	F (5, 315) = 4,789	P=0,0003
**Interaction: Time x Subjects**	1,044e-024	315	3,313e-027		
**Interaction: Place x Subjects**	53742	63	853		
**Subjects**	4,768e-025	63	7,568e-027		
**Residual**	108860	315	345,6		

Supp. Figure 1 (CSORC) – Two-way Repeated Measures ANOVA (Time as repeated measures)

Source of Variation	% of total variation	p value	p value summary	Significant?
**Interaction**	0,8434	0,7015	ns	No
**Time**	0,3881	0,9038	ns	No
**Sex**	0,5318	0,2184	ns	No
**Subject**	19,22	0,1554	ns	No

ANOVA table	SS	DF	MS	F (DFn, DFd)	p value
**Interaction**	174,5	5	34,91	F (5, 280) = 0,5980	P=0,7015
**Time**	80,31	5	16,06	F (4,264, 238,8) = 0,2752	P=0,9038
**Sex**	110,1	1	110,1	F (1, 56) = 1,549	P=0,2184
**Subject**	3978	56	71,04	F (56, 280) = 1,217	P=0,1554
**Residual**	16344	280	58,37		

Supp. Figure 1 (w1118) – Two-way Repeated Measures ANOVA (Time as repeated measures)

Source of Variation	% of total variation	p value	p value summary	Significant?
**Interaction**	0,8291	0,6913	ns	No
**Time**	1,456	0,3720	ns	No
**Sex**	0,05594	0,6516	ns	No
**Subject**	16,30	0,4793	ns	No

ANOVA table	SS	DF	MS	F (DFn, DFd)	p value
**Interaction**	281,1	5	56,23	F (5, 300) = 0,6114	P=0,6913
**Time**	493,7	5	98,75	F (4,325, 259,5) = 1,074	P=0,3720
**Sex**	18,97	1	18,97	F (1, 60) = 0,2059	P=0,6516
**Subject**	5526	60	92,10	F (60, 300) = 1,001	P=0,4793
**Residual**	27589	300	91,96		

Supp. Figure 2B – Two-way Repeated Measures ANOVA (repeated measures by both factors)

Source of Variation	% of total variation	P value	P value summary	Significant?
**Trial**	1,981	0,0419	*	Yes
**Before-After**	1,34	0,0002	***	Yes
**Interaction: Trial x Before-After**	0,1061	0,9110	ns	No
**Interaction: Trial x Subjects**	48,27			
**Interaction: Before-After x Subjects**	4,942			
**Subjects**	23,39			

ANOVA table	SS	DF	MS	F (DFn, DFd)	P value
**Trial**	819,5	5	163,9	F (5, 285) = 2,34	P=0,0419
**Before-After**	554,1	1	554,1	F (1, 57) = 15,45	P=0,0002
**Interaction: Trial x Before-After**	43,88	5	8,775	F (5, 285) = 0,3028	P=0,9110
**Interaction: Trial x Subjects**	19965	285	70,05		
**Interaction: Before-After x Subjects**	2044	57	35,86		
**Subjects**	9676	57	169,8		
**Residual**	8259	285	28,98		

Supp. Figure 2C – Two-way Repeated Measures ANOVA (repeated measures by both factors)

Source of Variation	% of total variation	P value	P value summary	Significant?
**Trial**	1,79	0,0429	*	Yes
**Before-After**	1,014	0,0001	***	Yes
**Interaction: Trial x Before-After**	0,319	0,3710	ns	No
**Interaction: Trial x Subjects**	46,95			
**Interaction: Before-After x Subjects**	3,582			
**Subjects**	28,34			

ANOVA table	SS	DF	MS	F (DFn, DFd)	P value
**Trial**	1385	5	277	F (5, 305) = 2,325	P=0,0429
**Before-After**	784,5	1	784,5	F (1, 61) = 17,26	P=0,0001
**Interaction: Trial x Before-After**	246,9	5	49,37	F (5, 305) = 1,081	P=0,3710
**Interaction: Trial x Subjects**	36340	305	119,1		
**Interaction: Before-After x Subjects**	2772	61	45,45		
**Subjects**	21932	61	359,5		
**Residual**	13935	305	45,69		

Supp. Figure 2D – Two-way Repeated Measures ANOVA (repeated measures by both factors)

Source of Variation	% of total variation	P value	P value summary	Significant?
**Trial**	1,527	0,0489	*	Yes
**Before-After**	0,0005875	0,9303	ns	No
**Interaction: Trial x Before-After**	0,1443	0,8631	ns	No
**Interaction: Trial x Subjects**	38,55			
**Interaction: Before-After x Subjects**	4,342			
**Subjects**	33,72			

ANOVA table	SS	DF	MS	F (DFn, DFd)	P value
**Trial**	1079	5	215,8	F (5, 285) = 2,258	P=0,0489
**Before-After**	0,4152	1	0,4152	F (1, 57) = 0,007712	P=0,9303
**Interaction: Trial x Before-After**	102	5	20,4	F (5, 285) = 0,3789	P=0,8631
**Interaction: Trial x Subjects**	27246	285	95,6		
**Interaction: Before-After x Subjects**	3069	57	53,84		
**Subjects**	23836	57	418,2		
**Residual**	15348	285	53,85		

Supp. Figure 3A – Pearson's Correlation

Pearson r

**r**	0,5007
**95% confidence interval**	0,4176 to 0,5756
**R squared**	0,2507

P value

**P (two-tailed)**	<0,0001
**P value summary**	****
**Significant? (alpha = 0.05)**	Yes
**Number of XY Pairs**	348

Supp. Figure 3B – Pearson's Correlation

Pearson r

**r**	0,5586
**95% confidence interval**	0,4845 to 0,6248
**R squared**	0,3121

P value

**P (two-tailed)**	<0,0001
**P value summary**	****
**Significant? (alpha = 0.05)**	Yes
**Number of XY Pairs**	372

Supp. Figure 3C – Pearson's Correlation

Pearson r

**r**	0,476
**95% confidence interval**	0,3904 to 0,5535
**R squared**	0,2266

P value

**P (two-tailed)**	<0,0001
**P value summary**	****
**Significant? (alpha = 0.05)**	Yes
**Number of XY Pairs**	348

Supp. Figure 4A – One-way ANOVA

ANOVA summary	
**F**	8,945
**P value**	0,0002
**P value summary**	***
**Significant diff. among means (P < 0.05)?**	Yes
**R square**	0,09275

ANOVA table	SS	DF	MS	F (DFn, DFd)	p value
**Treatment (between columns)**	0,7349	2	0,3675	F (2, 175) = 8,945	P=0,0002
**Residual (within columns)**	7,189	175	0,04108		
**Total**	7,923	177			

Holm-Sidak's multiple comparisons	Mean Diff	Significant?	Summary	Adjusted p value
**CSORC vs. w1118**	-0,1429	Yes	***	0,0005
**CSORC vs. Fragile X**	-0,1299	Yes	**	0,0014
**w1118 vs. Fragile X**	0,01294	No	ns	0,7272

Test details	Mean 1	Mean 2	Mean Diff	SE of diff	n1	n2	t	DF
**CSORC vs. w1118**	0,167	0,3099	-0,1429	0,03702	58	62	3,859	175
**CSORC vs. Fragile X**	0,167	0,297	-0,1299	0,03764	58	58	3,453	175
**w1118 vs. Fragile X**	0,3099	0,297	0,01294	0,03702	62	58	0,3495	175

Supp. Figure 4B – One-way ANOVA

ANOVA summary	
**F**	17,24
**P value**	<0,0001
**P value summary**	****
**Significant diff. among means (P < 0.05)?**	Yes
**R square**	0,1646

ANOVA table	SS	DF	MS	F (DFn, DFd)	p value
**Treatment (between columns)**	4,465	2	2,233	F (2, 175) = 17,24	P<0,0001
**Residual (within columns)**	22,67	175	0,1295		
**Total**	27,13	177			

Holm-Sidak's multiple comparisons	Mean Diff	Significant?	Summary	Adjusted p value
**CSORC vs. w1118**	-0,3855	Yes	****	<0,0001
**CSORC vs. Fragile X**	-0,2172	Yes	**	0,0028
**w1118 vs. Fragile X**	0,1683	Yes	*	0,0113

Test details	Mean 1	Mean 2	Mean Diff	SE of diff	n1	n2	t	DF
**CSORC vs. w1118**	0,07171	0,4572	-0,3855	0,06574	58	62	5,863	175
**CSORC vs. Fragile X**	0,07171	0,2889	-0,2172	0,06683	58	58	3,25	175
**w1118 vs. Fragile X**	0,4572	0,2889	0,1683	0,06574	62	58	2,559	175
