# Supplementary material for: A simple high-throughput method for automated detection of Drosophila melanogaster light-dependent behaviours
Source: BMC Biol. 2022 Dec 17;20:283. doi: 10.1186/s12915-022-01476-z (PMC9758938; doi:10.1186/s12915-022-01476-z)
Supplement: Supplementary file 6 — Additional file 6. Codes and Data. MATLAB codes and GraphPad Prism files. [file 12915_2022_1476_MOESM6_ESM.zip › Supplementary Material/DISCO codes/NeoPixel_Add-On_Library_for_Arduino.zip!/html/NeoPixelDocAddon.html]

addon 

# addon

Connect to Adafruit NeoPixel strip.

## Contents

- Syntax
- Description
- Input Arguments
- Name-Value Pair Arguments
- Properties
- Output Arguments
- Examples
- See Also

## Syntax

neostrip = addon(a, 'Adafruit/NeoPixel', DataPin, NumPixels)

neostrip = addon(a, 'Adafruit/NeoPixel', DataPin, NumPixels, 'NeoPixelType', NeoPixelType)

## Description

neostrip = addon(a, 'Adafruit/NeoPixel', DataPin, NumPixels) creates the connection to an Adafruit NeoPixel strip.

neostrip = addon(a, 'Adafruit/NeoPixel', DataPin, NumPixels, 'NeoPixelType', Name, Value) specifies additional options with one or more Name,Value pair arguments.

## Input Arguments

a - Arduino® hardware connection, specified as an arduino object.

'Adafruit/NeoPixel' - Adafruit NeoPixel library name.

DataPin - Arduino pin number to communicate with Adafruit NeoPixel.

NumPixels - Number of pixels on Adafruit NeoPixel.

## Name-Value Pair Arguments

Specify optional comma-separated pairs of Name,Value arguments. Name is the argument name and Value is the corresponding value. Name must appear inside single quotes (' '). You can specify several name and value pair arguments in any order as Name1,Value1,.., NameN,ValueN.

Use Name,Value arguments to specify writable properties NeoPixelType and Brightness during object creation.

For example, neostrip = addon(a, 'Adafruit/NeoPixel', 'D6', 30, 'NeoPixelType', 'BGR', 'Brightness', 0.4) creates a NeoPixel connection with NeoPixelType set to BGR and Brightness set to 40%.

## Properties

A NeoPixel object consists of the following properties, Pins, NumPixels, NeoPixelType and Brightness, that can be set during object creation.

|  |  |
| --- | --- |
| Pins | The Arduino pin used for communication with the Adafruit NeoPixel strip, specified as a string or character vector.   This is a read-only property and can be set only during object creation. |
| NumPixels | Number of pixels on Adafruit NeoPixel strip, specified as a numeric value.   This is a read-only property and can be set only during object creation. |
| NeoPixelType | The type of NeoPixel being used, specified as a string or character vector.   For RGB strip, any permutation of R, G and B is a valid input. Similarly, for RGBW any permutation of R, G, B, and W is valid.   The default value for NeoPixelType is 'GRB'.   This is a read-only property and can be set only during object creation. |
| Brightness | The brightness value for NeoPixel strip, specified as a numeric value between 0 to 1.   The default brightness is set to 0.25 |

## Output Arguments

neostrip - Adafruit NeoPixel connection, returned as a NeoPixel object.

## Examples

**Create an Adafruit NeoPixel Connection**

Create an Arduino object, and then create the NeoPixel object.

```
a = arduino('COM10', 'Uno', 'Libraries', 'Adafruit/NeoPixel')
neostrip = addon(a, 'Adafruit/NeoPixel', 'D6', 30)
```

```
a = 

  arduino with properties:

                    Port: 'COM10'
                   Board: 'Uno'
           AvailablePins: {'D2-D13', 'A0-A5'}
               Libraries: {'Adafruit/NeoPixel'}


neostrip = 

  NeoPixel with properties:

               Pins: 'D6'
          NumPixels:  30
       NeoPixelType: 'GRB'
         Brightness:  0.250
```

## See Also

arduino | readColor | writeColor

Published with MATLAB® R2017a
