# Supplementary material for: A simple high-throughput method for automated detection of Drosophila melanogaster light-dependent behaviours
Source: BMC Biol. 2022 Dec 17;20:283. doi: 10.1186/s12915-022-01476-z (PMC9758938; doi:10.1186/s12915-022-01476-z)
Supplement: Supplementary file 6 — Additional file 6. Codes and Data. MATLAB codes and GraphPad Prism files. [file 12915_2022_1476_MOESM6_ESM.zip › Supplementary Material/DISCO codes/NeoPixel_Add-On_Library_for_Arduino.zip!/html/NeoPixelDocReadColor.html]

readColor 

# readColor

Read the color(s) of one or more pixels.

## Contents

- Syntax
- Description
- Input Arguments
- Output Arguments
- Examples
- See Also

## Syntax

colors = readColor(neostrip)

colors = readColor(neostrip, indices)

## Description

colors = readColor(neostrip) reads the colors of all pixels on the NeoPixel strip.

colors = readColor(neostrip, indices) reads the color(s) of one or more pixels specified by indices parameter on the NeoPixel strip.

## Input Arguments

neostrip - Adafruit NeoPixel connection, specified as a NeoPixel object.

indices - Array of pixel indices.

## Output Arguments

colors - Color(s) represented as N-by-3 or N-by-4 array with columns representing RGB or RGBW normalized intensities respectively.

## Examples

**Read colors**

Create an Arduino® object, and create the NeoPixel object.

```
a = arduino('COM10', 'Uno', 'libraries', 'Adafruit/NeoPixel')
neostrip = addon(a, 'Adafruit/NeoPixel', 'D6', 7)
```

```
a = 

  arduino with properties:

                    Port: 'COM10'
                   Board: 'Uno'
           AvailablePins: {'D2-D13', 'A0-A5'}
               Libraries: {'Adafruit/NeoPixel'}


neostrip = 

  NeoPixel with properties:

               Pins: 'D6'
          NumPixels:  7
       NeoPixelType: 'GRB'
         Brightness:  0.250
```

**Write color to strip**

Use rand function to generate random colors for the pixels and write them using writeColor function.

```
randColors = rand(7, 3)
writeColor(neostrip, 1:7, randColors);
```

```
randColors =

    0.8147    0.5469    0.8003
    0.9058    0.9575    0.1419
    0.1270    0.9649    0.4218
    0.9134    0.1576    0.9157
    0.6324    0.9706    0.7922
    0.0975    0.9572    0.9595
    0.2785    0.4854    0.6557
```

**Read the colors of all pixels**

```
colors = readColor(neostrip)
```

```
colors =

    0.8147    0.5469    0.8003
    0.9058    0.9575    0.1419
    0.1270    0.9649    0.4218
    0.9134    0.1576    0.9157
    0.6324    0.9706    0.7922
    0.0975    0.9572    0.9595
    0.2785    0.4854    0.6557
```

**Read the colors of selected pixels**

Read the color of the first pixel.

```
colors = readColor(neostrip, 1)
```

```
colors =

    0.8147    0.5469    0.8003
```

Read the colors of the first, third, and fifth pixels.

```
colors = readColor(neostrip, [1, 3, 5])
```

```
colors =

    0.8147    0.5469    0.8003
    0.1270    0.9649    0.4218
    0.6324    0.9706    0.7922
```

## See Also

addon | writeColor

Published with MATLAB® R2017a
