# Supplementary material for: A simple high-throughput method for automated detection of Drosophila melanogaster light-dependent behaviours
Source: BMC Biol. 2022 Dec 17;20:283. doi: 10.1186/s12915-022-01476-z (PMC9758938; doi:10.1186/s12915-022-01476-z)
Supplement: Supplementary file 6 — Additional file 6. Codes and Data. MATLAB codes and GraphPad Prism files. [file 12915_2022_1476_MOESM6_ESM.zip › Supplementary Material/DISCO codes/NeoPixel_Add-On_Library_for_Arduino.zip!/html/NeoPixelDocWriteColor.html]

writeColor 

# writeColor

Write the color(s) to one or more pixels.

## Contents

- Syntax
- Description
- Input Arguments
- Examples
- See Also

## Syntax

writeColor(nstrip, colors)

writeColor(nstrip, indices, colors)

writeColor(nstrip, indices1, colors1, indices2, colors2, ..., indicesN, colorsN)

## Description

writeColor(nstrip, colors) writes the specified colors to all pixels on the given NeoPixel strip. A single color can be specified, to set the entire strip to the specified color. A N x 3 (RGB) or N x 4 (RGBW) array can be specified to write different colors to all pixels at once. Note that the value of N in the input the colors array must match the NumPixels property.

writeColor(nstrip, indices, colors) writes the specified colors to the specified pixels. The inputs indices and colors must have compatible dimensions, that is if indices is a 1 x N array, then the colors could be either single value or a N x 3 (RGB) or N x 4 (RGBW) array.

writeColor(nstrip, index1, color1, index2, color2, ..., indexN, colorN) writes the specified colors to corresponding pixels on a NeoPixel strip in a single instruction. Each index is a single numeric value and color specifies the corresponding color value of the pixel.

writeColor(nstrip, indices1, colors1, indices2, colors2, ..., indicesN, colorsN) writes the specified colors to corresponding pixels on a NeoPixel strip in a single instruction. Indices can be 1 x N array while corresponding colors can be either a single color or N x 3 (RGB) or N x 4 (RGBW) array.

## Input Arguments

nstrip - Adafruit NeoPixel connection, specified as a NeoPixel object.

indices - Array of pixel indices to write color.

colors - Color of the pixel. The colors can be specified in three ways. First is using ColorSpec, which allows verbose specification of color like 'red', 'green', etc. Second is to provide an explicit N x 3 for RGB strip or N x 4 for RGBW strip, where N is the number of pixels. Third is to specify the value off, which turns all the pixels on the NeoPixel strip off.

**Note:** Whenever colors is specified as an array of R, G, B and/or W components, the order is expected to be [R, G, B] or [R, G, B, W] irrespective of the NeoPixelType of the strip.

## Examples

**Initialization**

Create an Arduino® object, and create the NeoPixel object.

```
a = arduino('COM10', 'Uno', 'libraries', 'Adafruit/NeoPixel')
nstrip = addon(a, 'Adafruit/NeoPixel', 'D6', 30)
```

```
a = 

  arduino with properties:

                    Port: 'COM10'
                   Board: 'Uno'
           AvailablePins: {'D2-D13', 'A0-A5'}
               Libraries: {'Adafruit/NeoPixel'}


nstrip = 

  NeoPixel with properties:

               Pins: 'D6'
          NumPixels:  30
       NeoPixelType: 'GRB'
         Brightness:  0.250
```

**Set all pixels to Red**

```
% Specifying color using ColorSpec
writeColor(nstrip, 'red');

% Specifying color explicitly using R, G, B components
writeColor(nstrip, [1, 0, 0]);
```

**Turn off all pixels**

```
writeColor(nstrip, 'off');
```

**Set color of selected pixel to Blue**

```
% Writing color to single pixel
writeColor(nstrip, 1, 'blue');

% Writing color to multiple pixels
writeColor(nstrip, 1:4, [0, 0, 1]);
```

**Set color of first pixel to Red and second pixel to Green**

```
% Using syntax: writeColor(nstrip, indices, colors)
writeColor(nstrip, [1, 2], [1, 0, 0; 0, 1, 0]);

% Using syntax: writeColor(nstrip, indices1, color1, indices2, color2, ...);
writeColor(nstrip, 1, [1, 0, 0], 2, [0, 1, 0]);
```

## See Also

addon | readColor

Published with MATLAB® R2017a
