# Supplementary material for: A simple high-throughput method for automated detection of Drosophila melanogaster light-dependent behaviours
Source: BMC Biol. 2022 Dec 17;20:283. doi: 10.1186/s12915-022-01476-z (PMC9758938; doi:10.1186/s12915-022-01476-z)
Supplement: Supplementary file 6 — Additional file 6. Codes and Data. MATLAB codes and GraphPad Prism files. [file 12915_2022_1476_MOESM6_ESM.zip › Supplementary Material/DISCO codes/NeoPixel_Add-On_Library_for_Arduino.zip!/html/NeoPixelExample_Basic.html]

Basic Control of Adafruit NeoPixel 

# Basic Control of Adafruit NeoPixel

This example shows how to use the MATLAB® Support Package for Arduino® Hardware to control an Adafruit NeoPixel RGB LED strip.

## Contents

- Hardware Requirements
- Hardware Setup
- Create NeoPixel Object
- Write Color to NeoPixel
- Read Colors from Pixel
- Update and Read Brightness
- Clean Up

## Hardware Requirements

- Arduino Uno board
- Adafruit NeoPixel RGB strip with at least 12 pixels
- External 5V power supply
- 1000uF capacitor

## Hardware Setup

- Connect DIN pin on NeoPixel strip to digital pin D6 on Arduino board.
- Connect VCC, GND pin to external 5V power supply (Recommended current rating 2 Amp for this example).
- Connect 1000uF capacitor between the power supply terminals to supress voltage spikes.

## Create NeoPixel Object

**Create Arduino object**

```
a = arduino('COM10', 'Uno', 'Libraries', 'Adafruit/NeoPixel')
```

```
a = 

  arduino with properties:

                    Port: 'COM10'
                   Board: 'Uno'
           AvailablePins: {'D2-D13', 'A0-A5'}
               Libraries: {'Adafruit/NeoPixel'}
```

**Create NeoPixel object**

The following instantiation of a NeoPixel object uses the default NeoPixelType of GRB and Brightness value of 25%.

```
neostrip = addon(a, 'Adafruit/NeoPixel', 'D6', 12)
```

```
neostrip = 

  NeoPixel with properties:

               Pins: 'D6'
          NumPixels:  12
       NeoPixelType: 'GRB'
         Brightness:  0.250
```

## Write Color to NeoPixel

It is possible to write the color of a pixel in several ways using the writeColor method. The following examples demonstrate the different ways to use the method. It must be noted that irrespective of NeoPixelType of the strip, input color order must be [R, G, B].

**Write color to a single pixel**

The first command uses ColorSpec color to set first pixel to green. In the second command, the second pixel is set to blue color by providing 1x3 array of [R, G, B].

```
writeColor(neostrip, 1, 'green');       % Pixel -> Green
writeColor(neostrip, 1, [0 0 1]);       % Pixel -> Blue
```

**Write same color to multiple pixels**

The following commands writes the same color of multiple pixels; 3, 4 and 5, to red in single command. It is possible to input color using 1x3 or 3x3 array as demonstrated.

```
writeColor(neostrip, 3:5, 'red');       % Pixel -> Red
writeColor(neostrip, [6 7 8], [0 1 0]); % Pixels -> Green
writeColor(neostrip, [9 10 11], [0 0 1; 0 0 1; 0 0 1]); % Pixels -> ReBlued
```

**Write different colors to different pixels**

The following commands writes different colors to different pixels in single command.

```
writeColor(neostrip, 6, 'magenta', 7, 'yellow');   % Pixel -> [Magenta, Yellow]
writeColor(neostrip, [8 9 10], [1 0 0; 0 1 0; 0 0 1]); % Pixels -> [Red, Green, Blue]
```

**Note**: Writing multiple pixels in a single command is faster than calling writeColor separately for each individual pixel.

## Read Colors from Pixel

**Read Color of all pixels**

```
value = readColor(neostrip)
```

```
value =

     0     0     1
     0     0     0
     1     0     0
     1     0     0
     1     0     0
     1     0     1
     1     1     0
     1     0     0
     0     1     0
     0     0     1
     0     0     1
     0     0     0
```

**Read color of a single pixel**

```
value = readColor(neostrip, 1)
```

```
value =

     0     0     1
```

**Read color of 3 pixels**

```
value = readColor(neostrip, [5 6 7])
```

```
value =

     1     0     0
     1     0     1
     1     1     0
```

## Update and Read Brightness

**Read Brightness**

```
brightness = neostrip.Brightness
```

```
brightness =

    0.2500
```

**Update Brightness**

The following code updates the brightness of all the pixels on the NeoPixel strip. This command does not affect the color of any pixel. Only the intensity is changed.

```
neostrip.Brightness = 0.1
```

```
neostrip = 

  NeoPixel with properties:

               Pins: 'D6'
          NumPixels:  12
       NeoPixelType: 'GRB'
         Brightness:  0.100
```

## Clean Up

```
clear neostrip
clear a
```

Copyright 2017 The MathWorks, Inc.  
Published with MATLAB® R2017a
