# Supplementary material for: A simple high-throughput method for automated detection of Drosophila melanogaster light-dependent behaviours
Source: BMC Biol. 2022 Dec 17;20:283. doi: 10.1186/s12915-022-01476-z (PMC9758938; doi:10.1186/s12915-022-01476-z)
Supplement: Supplementary file 6 — Additional file 6. Codes and Data. MATLAB codes and GraphPad Prism files. [file 12915_2022_1476_MOESM6_ESM.zip › Supplementary Material/DISCO codes/NeoPixel_Add-On_Library_for_Arduino.zip!/html/NeoPixelExample_Gradient.html]

Control Two Adafruit NeoPixels Simultaneously 

# Control Two Adafruit NeoPixels Simultaneously

This example shows how to use the MATLAB® Support Package for Arduino® Hardware to control two Adafruit NeoPixel LED Strips simultaneously.

## Contents

- Hardware Requirements
- Hardware Setup
- Create Arduino Instance
- Write the Colors to Produce Gradient Effect
- Clean Up

## Hardware Requirements

- Arduino Uno board
- Adafruit NeoPixel RGB Strip with 30 pixels
- Adafruit NeoPixel RGBW Strip with 60 pixels
- External 5V power supply
- 1000uF capacitor

## Hardware Setup

- Connect DIN pin on NeoPixel RGB strip to D6 on Arduino board.
- Connect DIN pin on NeoPixel RGBW strip to D7 on Arduino board.
- Connect VCC, GND pin to external 5V power supply (recommended current rating of 5 Amp or more for this example).
- Connect 1000uF capacitor between the power supply terminals to supress voltage spikes.

## Create Arduino Instance

**Create Arduino object**

```
a = arduino('COM10', 'Uno', 'Libraries', 'Adafruit/NeoPixel')
```

```
a = 

  arduino with properties:

                    Port: 'COM10'
                   Board: 'Uno'
           AvailablePins: {'D2-D13', 'A0-A5'}
               Libraries: {'Adafruit/NeoPixel'}
```

**Create NeoPixel objects**

```
neostrip1 = addon(a, 'Adafruit/NeoPixel', 'D6', 30, 'NeoPixelType', 'GRB')
neostrip2 = addon(a, 'Adafruit/NeoPixel', 'D7', 60, 'NeoPixelType', 'GRBW')
```

```
neostrip1 = 

  NeoPixel with properties:

               Pins: 'D6'
          NumPixels:  30
       NeoPixelType: 'GRB'
         Brightness:  0.250


neostrip2 = 

  NeoPixel with properties:

               Pins: 'D7'
          NumPixels:  60
       NeoPixelType: 'GRBW'
         Brightness:  0.250
```

## Write the Colors to Produce Gradient Effect

**Define desired colorMaps**

The colormap returns an array of RGB values that represent gradually changing colors from one color to another. The use of the hsv function creates a rainbow effect, while the lines function has various contrast colors. Note that we must append zeros to colors2 because the second strip is a RGBW strip, while the colormap function returns RGB values only.

```
colors1 = hsv(255);
colors2 = [lines(255), zeros([255, 1])];
```

**Iteratively update colors**

As the number of pixels (30 or 60) is less than the number of selected colors (i.e. 255), using circshift we can show all the colors from colors1 and colors2 on respective NeoPixel strips by circularly shifting the color window.

```
for idx = 1:25
    % Perform a circular shift the colors
    colors1 = circshift(colors1, [1, 0]);
    colors2 = circshift(colors2, [1, 0]);

    % Write colors to neoPixel strips
    writeColor(neostrip1, 1:neostrip1.NumPixels, colors1(1:neostrip1.NumPixels, :));
    writeColor(neostrip2, 1:neostrip2.NumPixels, colors2(1:neostrip2.NumPixels, :));
end
```

## Clean Up

```
clear neostrip2
clear neostrip1
clear a
```

Copyright 2017 The MathWorks, Inc.  
Published with MATLAB® R2017a
