# Supplementary material for: A simple high-throughput method for automated detection of Drosophila melanogaster light-dependent behaviours
Source: BMC Biol. 2022 Dec 17;20:283. doi: 10.1186/s12915-022-01476-z (PMC9758938; doi:10.1186/s12915-022-01476-z)
Supplement: Supplementary file 6 — Additional file 6. Codes and Data. MATLAB codes and GraphPad Prism files. [file 12915_2022_1476_MOESM6_ESM.zip › Supplementary Material/DISCO codes/NeoPixel_Add-On_Library_for_Arduino.zip!/html/NeoPixelExample_Sequencer.html]

Display Sequencing Effects on Adafruit NeoPixel 

# Display Sequencing Effects on Adafruit NeoPixel

This example shows how to use the MATLAB® Support Package for Arduino® Hardware to implement sequence lighting effects on an Adafruit NeoPixel RGB LED Strip.

## Contents

- Hardware Requirements
- Hardware Setup
- Create Objects
- Sequence Solo Mover
- Christmas Solo Mover
- Clean Up

## Hardware Requirements

- Arduino Uno board
- Adafruit NeoPixel RGB Strip with 30 pixels
- External 5V power supply
- 1000uF capacitor

## Hardware Setup

- Connect DIN pin on NeoPixel RGB strip to D6 on Arduino board.
- Connect VCC, GND pin to external 5V power supply (recommended current rating 2 Amp for this example).
- Connect 1000uF capacitor between the power supply terminals to supress voltage spikes.

## Create Objects

**Create Arduino object**

```
a = arduino('COM10', 'Uno', 'Libraries', 'Adafruit/NeoPixel')
```

```
a = 

  arduino with properties:

                    Port: 'COM10'
                   Board: 'Uno'
           AvailablePins: {'D2-D13', 'A0-A5'}
               Libraries: {'Adafruit/NeoPixel'}
```

**Create NeoPixel object**

The following instantiation of a NeoPixel object uses the default NeoPixelType of GRB and Brightness value of 25%.

```
neostrip = addon(a, 'Adafruit/NeoPixel', 'D6', 30)
```

```
neostrip = 

  NeoPixel with properties:

               Pins: 'D6'
          NumPixels:  30
       NeoPixelType: 'GRB'
         Brightness:  0.250
```

## Sequence Solo Mover

This example shows how to produce a sequential light effect by moving a color from the start of the strip to its end.

**Initialize the first pixel to show cyan color**

```
writeColor(neostrip, 1, 'cyan');
```

**Iteratively Shift color**

The color is shifted by turning off the presently lit pixel and writing color to next pixel in the sequence.

```
for idx = 2:neostrip.NumPixels
    writeColor(neostrip, idx-1, 'off', idx, 'cyan');
    pause(0.2);   % Introduce delay of 200ms to achieve the effect
end
```

**Turn off the last pixel**

```
writeColor(neostrip, neostrip.NumPixels, 'off');
```

## Christmas Solo Mover

This example shows how to produce a Christmas lighting effect by lighting alternate pixels with red and green color.

**Initialize the first pixel to show red color**

```
writeColor(neostrip, 1, 'red');
```

**Iteratively write colors to pixels**

Write all even pixels green and all odd pixels red

```
for idx = 2:neostrip.NumPixels
    if mod(idx, 2) == 0
        writeColor(neostrip, idx, 'green');
    else
        writeColor(neostrip, idx, 'red');
    end
    pause(0.2)  % Introduce delay of 200ms to achieve the effect
end
```

## Clean Up

```
clear neostrip
clear a
```

Copyright 2017 The MathWorks, Inc.  
Published with MATLAB® R2017a
