# Supplementary material for: A simple high-throughput method for automated detection of Drosophila melanogaster light-dependent behaviours
Source: BMC Biol. 2022 Dec 17;20:283. doi: 10.1186/s12915-022-01476-z (PMC9758938; doi:10.1186/s12915-022-01476-z)
Supplement: Supplementary file 6 — Additional file 6. Codes and Data. MATLAB codes and GraphPad Prism files. [file 12915_2022_1476_MOESM6_ESM.zip › Supplementary Material/DISCO codes/NeoPixel_Add-On_Library_for_Arduino.zip!/html/NeoPixelExample_Thermometer.html]

Temperature Visualization on Adafruit NeoPixel 

# Temperature Visualization on Adafruit NeoPixel

This example shows how to use the MATLAB® Support Package for Arduino® Hardware to control an Adafruit NeoPixel RGB LED Strip to visualize current temperature using TMP36 temperature sensor.

## Contents

- Hardware Requirements
- Hardware Setup
- Define Configuration Variables
- Create NeoPixel Object
- Temperature-based Lighting
- Clean Up

## Hardware Requirements

- Arduino Uno board
- Adafruit NeoPixel RGB Strip with 30 pixels
- TMP36 temperature sensor
- External 5V power supply
- 1000uF capacitor

## Hardware Setup

- Connect DIN pin on NeoPixel RGB strip to D6 on Arduino board.
- Connect VCC, GND pin to external 5V power supply (recommended current rating 2 Amp for up to 60 pixels).
- Connect 1000uF capacitor between the power supply terminals to supress voltage spikes.
- Connect VOUT pin on TMP36 sensor to A5 on Arduino board.
- Connect Vs, GND pins of TMP36 to VCC and GND on Arduino board.

## Define Configuration Variables

Define the minimum and maximum temperature to visualize on the NeoPixel strip. The minimum temperature corresponds to no pixels lit while maximum temperature corresponds to all 30 pixels lit, as shown in the following figure.

```
% Range of temperature (in celsius) to show on NeoPixel Strip
minTemp = 22;
maxTemp = 28;
```

## Create NeoPixel Object

**Create Arduino object**

```
a = arduino('COM10', 'Uno', 'Libraries', 'Adafruit/NeoPixel')
```

```
a = 

  arduino with properties:

                    Port: 'COM10'
                   Board: 'Uno'
           AvailablePins: {'D2-D13', 'A0-A5'}
               Libraries: {'Adafruit/NeoPixel'}
```

**Create NeoPixel object**

```
% Using default brightness value
neostrip = addon(a, 'Adafruit/NeoPixel', 'D6', 30, 'NeoPixelType', 'GRB')
```

```
neostrip = 

  NeoPixel with properties:

               Pins: 'D6'
          NumPixels:  30
       NeoPixelType: 'GRB'
         Brightness:  0.250
```

## Temperature-based Lighting

The temperature-based lighting does the following:

- Read raw voltage output from TMP36 sensor
- Convert voltage to temperature and plot it using animatedline
- Scale temperature such that 22 C results in 0 pixels lit and 28 C results in all 30 pixels lit.
- Use colormap to show how hot it is on NeoPixel strip

**Create an animated line**

The animatedline function allows you to plot the current temperature live.

```
h = animatedline('MaximumNumPoints', Inf);
ylim([minTemp, maxTemp]),
xlabel('Time-steps'),
ylabel('Temperature (Celsius)');
title('TMP36 Temperature vs. Time-steps')
```

**Define colors using colormap**

The cool function in colormap represents gradual change in color from blue to violet. In this example, blue color would represent low temperature while the violet would reprensent a high temperature.

```
colors = cool(30);
```

**Run the loop to read temperature and update the colors of the NeoPixel strip**

The temperature is read from the TMP36 sensor connected to pin A5 on the arduino. The first line reads the raw analog input a value from this pin represented as value between 0V to 5V. This value is converted to temperature using the formula provided in the TMP36 datasheet.

This value is first clamped between minTemp and maxTemp defined above, and then scaled accordingly to determine the number of pixels that represent the measured temperature. Finally, the value is written to the NeoPixel strip and the realtime plot is updated.

```
for idx = 1:100
    % Read temperature
    temp = (readVoltage(a, 'A5') - 0.5) * 100;  % Formula adopted from TMP36 datasheet

    % Update the animatedline for plotting
    addpoints(h, idx, temp);

    % Clamp temperature
    if temp < minTemp
        temp = minTemp;
    elseif temp > maxTemp
        temp = maxTemp;
    end

    % Compute number of pixels to turn on
    onPixels = round((temp - minTemp) / (maxTemp - minTemp) * neostrip.NumPixels);

    % Send command to NeoPixel Strip
    writeColor(neostrip, 1:neostrip.NumPixels, [colors(1:onPixels, :); zeros(neostrip.NumPixels - onPixels, 3)]);

    % Update plot
    drawnow
end
```

## Clean Up

```
clear neostrip
clear a
```

Copyright 2017 The MathWorks, Inc.  
Published with MATLAB® R2017a
