# Supplementary material for: A simple high-throughput method for automated detection of Drosophila melanogaster light-dependent behaviours
Source: BMC Biol. 2022 Dec 17;20:283. doi: 10.1186/s12915-022-01476-z (PMC9758938; doi:10.1186/s12915-022-01476-z)
Supplement: Supplementary file 6 — Additional file 6. Codes and Data. MATLAB codes and GraphPad Prism files. [file 12915_2022_1476_MOESM6_ESM.zip › Supplementary Material/DISCO codes/NeoPixel_Add-On_Library_for_Arduino.zip!/html/NeoPixelFunclist.html]

Function Reference 

# Function Reference

The Adafruit NeoPixel add-on library for Arduino® has the following functions:

- addon - Connect to Adafruit NeoPixel strip.
- readColor - Read the color(s) of one or more pixels.
- writeColor - Write the color(s) of one or more pixels.

Published with MATLAB® R2017a
