# Supplementary material for: A simple high-throughput method for automated detection of Drosophila melanogaster light-dependent behaviours
Source: BMC Biol. 2022 Dec 17;20:283. doi: 10.1186/s12915-022-01476-z (PMC9758938; doi:10.1186/s12915-022-01476-z)
Supplement: Supplementary file 6 — Additional file 6. Codes and Data. MATLAB codes and GraphPad Prism files. [file 12915_2022_1476_MOESM6_ESM.zip › Supplementary Material/DISCO codes/NeoPixel_Add-On_Library_for_Arduino.zip!/html/NeoPixelGettingStarted.html]

Getting Started with NeoPixel Add-On Library for Arduino 

# Getting Started with NeoPixel Add-On Library for Arduino

Before you can use this add-on library in MATLAB®, you need to install the Adafruit NeoPixel Arduino® library. To install the Adafruit NeoPixel Arduino library:

1. Download the zip file from GitHub.
2. Unpackage the zip file into a local directory and rename the folder to Adafruit\_NeoPixel.
3. Move the Adafruit\_NeoPixel folder into the libraries folder inside your Arduino sketchbook folder:

- On Windows®, the default path is "Documents\Arduino\libraries\"
- On Mac®, the default path is "~/Documents/Arduino/libraries/"
- On Linux®, the default path is "/home/<username>/Arduino/libraries"

Published with MATLAB® R2017a
