# Supplementary material for: A simple high-throughput method for automated detection of Drosophila melanogaster light-dependent behaviours
Source: BMC Biol. 2022 Dec 17;20:283. doi: 10.1186/s12915-022-01476-z (PMC9758938; doi:10.1186/s12915-022-01476-z)
Supplement: Supplementary file 6 — Additional file 6. Codes and Data. MATLAB codes and GraphPad Prism files. [file 12915_2022_1476_MOESM6_ESM.zip › Supplementary Material/DISCO codes/NeoPixel_Add-On_Library_for_Arduino.zip!/html/NeoPixelTopFile.html]

NeoPixel Add-On Library for Arduino 

# NeoPixel Add-On Library for Arduino

NeoPixel add-on library for Arduino® enables you to use MATLAB® to communicate with an Adafruit NeoPixel connected to an Arduino board. Adafruit NeoPixel are individually addressable RGB or RGBW LEDs.

Getting Started with NeoPixel Add-On Library for Arduino

Functions

For more information on NeoPixel, visit Adafruit NeoPixel Guide.

Published with MATLAB® R2017a
